# Unveiling the link between phytoplankton molecular physiology and biogeochemical cycling via genome-scale modeling

**DOI:** 10.1126/sciadv.adq3593

**Published:** 2025-06-04

**Authors:** Antoine Régimbeau, Olivier Aumont, Chris Bowler, Lionel Guidi, George A. Jackson, Eric Karsenti, Laurent Memery, Alessandro Tagliabue, Damien Eveillard

**Affiliations:** ^1^ Nantes Université, Centrale Nantes, CNRS, LS2N, 2 rue de la Houssinière, 44322 Nantes, France.; ^2^ IRD-LOCEAN, IPSL, 4 place Jussieu, 75252 Paris, France.; ^3^ Institut de Biologie de l’ENS, Département de Biologie (IBENS), École Normale supérieure, CNRS, INSERM, Université PSL, 75005 Paris, France.; ^4^ Research Federation for the Study of Global Ocean Systems Ecology and Evolution GOSEE, CNRS FR2022/Tara Oceans, 3 rue Michel-Ange, 75016 Paris, France.; ^5^ Sorbonne Université, CNRS, Laboratoire d’Océanographie de Villefranche, LOV, Villefranche-sur-mer, France.; ^6^ Oceanography Department, Texas A&M University, 3146 TAMU, College Station, TX 77843, USA.; ^7^ Laboratoire des Sciences de l’Environnement Marin (LEMAR), Univ Brest, CNRS, IRD, Ifremer, IUEM, Plouzané, France.; ^8^ Department of Earth, Ocean and Ecological Sciences, University of Liverpool, Liverpool L69 3GP, UK.

## Abstract

Earth system models (ESMs) highly simplify their representation of biological processes, leading to major uncertainty in the impacts of climate change. Despite a growing understanding of molecular networks from genomic data, describing how changing phytoplankton physiology affects biogeochemical processes remains elusive. Here, we embed genome-scale models within a state-of-the-art ESM to deliver an integrated understanding of how gradients of nutrients modulate the molecular physiology of various plankton. In particular, when applied to *Prochlorococcus*, we find that glycogen and lipid management can be interpreted in terms of acclimation to different environments. Generalized to other phytoplankton such as the diatom *Thalassiosira*, we estimate the production of 39 metabolites that constitute hot spots of dissolved organic carbon described by their amount of carbon produced and their diversity of associated metabolites in ESMs. This modeling approach shows how genome scale–enabled ESMs have the potential to advance our understanding of microbial ecosystem functioning in ocean biogeochemical processes.

## Teaser

A genome-enabled ESM built on genomic data assesses physiological acclimation and biogeochemical effects through nutrient stress.

## INTRODUCTION

Earth system models (ESMs) are a powerful tool to study the future impact of climate change on the ocean (*1*). However, because of computational limitations (*2*, *3*), they need to simplify biology and biological processes, which limits our ability to understand and implement biological feedbacks on climate and biogeochemistry. For example, the net growth of an organism is described by a set of ordinary differential equations (*4*, *5*) involving nutrient uptake based on a scheme introduced by Monod (*6*) and later extended by Droop (*7*) through the use of cellular quotas. Following these approaches, more recent plankton functional type models (*2*, *8*) rely on extensive efforts to estimate a broad set of parameters that affects plankton functional diversity and describes traits critical to biogeochemical processes, such as the size or temperature of optimal growth. Efforts in modeling the macromolecular composition of phytoplankton aim to better represent their physiology (*9*). However, there is a fundamental disconnect between the biological underpinnings of today’s ESMs based on nutrient limitation or other phenotypical traits and the ever-growing gene- and genome-centered datasets that have emerged over recent years (*10*–*12*).

Using distinct approaches, two notable modeling studies have addressed the discrepancy between molecular functions and oceanic provinces. In 2017, Coles *et al.* (*10*) developed a trait-based model that harnessed omics data. This approach characterized omics-derived traits and simulated interactions between them, providing a computationally feasible representation of a community’s molecular functions in an oceanic environment. More recently, Casey *et al.* (*13*) focused on modeling *Prochlorococcus* communities along an Atlantic transect. Their work relies on extensive parameterization and optimization of genome-scale models (GSMs) using omics data. However, for it to be applied globally to all planktonic organisms, this approach demands computational power and out-of-reach data, including, but not limited to, the in situ molecular activity and concentration of modeled organisms under various environmental conditions and their response to temperature changes.

GSMs, developed primarily for bioengineering (*14*), offer an effective way to engage with growing biological datasets, as they use gene-protein-reaction associations to more comprehensively represent the metabolic potential of an organism as defined by its genetic material (*15*). GSMs consider a set of reactions organized into metabolic networks in which products of some reactions become substrates for others. The model’s genome-scale nature requires the description of many reactions, ranging from several hundreds for prokaryotes to thousands for eukaryotes. If environmental conditions are also incorporated, then GSMs can predict an organism’s growth rate, the production of auxiliary metabolites, or the metabolic pathways it uses (*16*). For example, they can be used to assess the diversity and magnitude of metabolite production (*17*) that contributes to the oceanic dissolved organic carbon (DOC) pool. While DOC represents one-quarter of photosynthesis-derived carbon on Earth, its prediction is difficult to assess via standard mechanistic models, as its metabolites are highly bioavailable and their production relies on understanding the entire plankton biocomplexity (*18*).

Here, we propose a modeling compromise that balances mechanistic complexity with computational efficiency. Through the connection of ESMs and GSMs, we simulate the growth of various organisms, study the acclimation of *Prochlorococcus*, and assess the organism’s potential contribution to the carbon pump through the production of dissolved organic compounds. These successful numerical experiments are a first attempt to bridge the gap between ESMs and the molecular understanding of organisms and hold great promise for advancing our understanding of microbial impact on vast ocean biogeochemical processes and improving current biotic predictions under different climate change scenarios (*19*).

## RESULTS

### Incorporating genome-scale knowledge into biogeochemical models

**Fig. 1.**
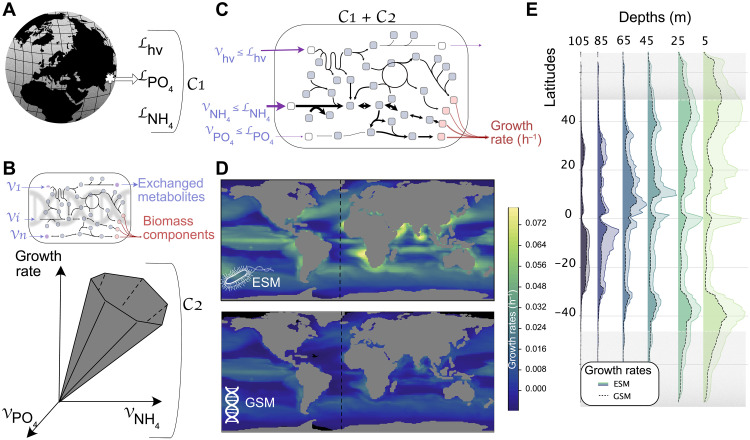
Illustration of the modeling combination between a GSM for *Prochlorococcus* MED4 and the NEMO-PISCES ESM and comparison between growth rates estimated from ESM and GSM. (**A**) ESMs predict global ocean biogeochemistry across space and time. Here, the ESM provides uptake fluxes of nutrients for each grid point for each modeled organism. In our framework, these uptake values are used as a set of constraints (C1) on the exchange reactions that feeds the GSM. (**B**) From a GSM, we defined a solution space embedding all possible fluxes that go through each network reaction. These fluxes satisfy the quasi–steady state assumption and other thermodynamic constraints. This set of constraints (C2) is defined as biotic constraints, and they affect inner reactions, as well as exchange reactions responsible for the uptake or secretion of nutrients and a biomass reaction simulating the growth of the organism (see appendix S1). (**C**) Abiotic constraints from the ESM and biotic constraints are combined to constrain the exchanged metabolite fluxes at each grid point of the global ocean. As a result, we can estimate the organismal growth rate and all feasible fluxes corresponding to a given environment as proposed by the ESM. (**D**) Description of growth rates at 5 m depth estimated from NEMO-PISCES picophytoplankton (top) and *Prochlorococcus* MED4 GSM (bottom). The dashed line shows the transect described in (E). (**E**) Distribution of respective growth rates across latitudes and depths at longitude of −24°. Gray areas indicate latitudes that do not allow *Prochlorococcus* MED4 growth because of thermal limits (temperatures < 10°C). The relationship between growth rates across space (above 500 m depth and in regions with temperatures above 10°C) and time shows a correlation coefficient $R^{2}=0.77$ and a slope of 0.45 (see fig. S5).

Simulating a GSM within an ESM requires solving several hundred equations at each grid point on Earth, which is currently computationally very demanding. In this work, we combined GSMs with the quota version of the Nucleus for European Modeling of the Ocean–Pelagic Interactions Scheme for Carbon and Ecosystem Studies (NEMO-PISCES) global ocean biogeochemical model (*20*), which is a classic example of a coupled ocean physicochemical-biological model embedded within an ESM used for climate change studies. NEMO-PISCES predicts the spatiotemporal distribution of three coarse-grained, cosmopolitan phytoplankton groups: picophytoplankton, nanophytoplankton, and diatoms. The ESM considers environmental conditions such as temperature, light, and a range of major macronutrients (Fig. 1A) for each group and uses these conditions to compute the associated group’s growth rate. We used the same conditions to calculate offline growth rates using GSMs over the annual cycle (Fig. 1, B and C, and see Materials and Methods for more details). We used a metabolic niche approach to address the computational complexity underlying the use of GSMs (*21*). This approach projects a species’ phenotype into a mathematical space driven by the availability of nutrients (see Fig. 1B for illustration and Materials and Methods for complete details). This abstraction describes the dependencies between growth rate and environmental conditions and allows us to explore cellular mechanisms originally described in the GSM.

Linking the environmental conditions produced by NEMO-PISCES with our numerical abstraction, we computed the growth rates of phytoplankton and the production of various metabolites by the modeled organisms. Our growth rate estimations were qualitatively comparable with NEMO-PISCES outputs, which have been extensively reviewed and used for biogeochemical predictions (*4*). *Prochlorococcus* MED4 GSM (*22*) qualitatively reproduced the patterns of average monthly NEMO-PISCES picophytoplankton growth rates both at the surface (Fig. 1D) and at different depths (Fig. 1E) over the global ocean (fig. S5). Our predictions also aligned with in situ data of the Atlantic Meridional Transect AMT13 (see appendix S4.2). To assess the applicability of our approach, we used it with other GSMs that are currently available for marine phytoplankton [i.e., *Synechococcus* sp. *PCC7002* (*23*), *Prochlorococcus* pangenome (*13*), *Thalassiosira pseudonana* (*24*), and *Phaeodactylum tricornutum* (*25*)]. Using the conditions computed by NEMO-PISCES for diatoms, we found a high correlation $R^{2}>0.76$ between NEMO-PISCES diatom growth and the GSM-based growth, indicating that the predictions were also accurate (fig. S13). Our framework can assess the comparison of environmental fitting between several species (see an approximation of competition between diatoms in fig. S14).

Nevertheless, we observed a quantitative mismatch between NEMO-PISCES and GSM growth rates. This was expected, as the GSMs only represented one strain of the corresponding NEMO-PISCES group [i.e., in situ abundance of the MED4 strain represents only about one-third of the total *Prochlorococcus* abundance (*26*)]. By adding other ecotypes (*26*, *27*) via the simulation of the *Prochlorococcus* pangenome (*13*) and *Synechococcus* sp. *PCC 7002* (*23*), we showed less cumulative error than each GSM taken individually (see appendix S5.4 for details), which strengthened our predictions. This leads us to think that, through a finer resolution of the community, we are reducing the inherent error due to the various parameterizations of the model.

### GSM-based predictions of *Prochlorococcus* MED4 in the global ocean

However, the strength of our approach lies not in reproducing NEMO-PISCES estimates but in exploiting organisms’ embedded biocomplexity through their GSMs to assess biogeography. For this purpose, we followed a similar assumption proposed in seminal studies that consider cyanobacterial molecular traits as a proxy for assessing ocean biome conditions [see Martiny *et al.* (*28*) for review]. Similar to investigating all transcripts of a given organism, GSMs can reveal any flux occurring through a metabolic process within an organism at the intracellular level—as long as it is defined within the metabolic network. Our simulations are thus not limited to growth rate estimates. They can be exploited to quantify the production of any metabolite represented in the GSM and investigate the corresponding pathways’ activity in response to environmental gradients. Unlike previous work that focuses on statistical description (*29*) or proteomic measurements (*30*) of resource limitation, we extended the use of GSMs and introduced the concept of “resource constraint.” This concept represents how the nutrient uptake variability affects the organism’s growth (see Materials and Methods for a mathematical definition and appendix S4.3 for a more detailed discussion). For a given nutrient, a low resource constraint means that a substantial amount of the nutrient can be used for processes other than growth, such as secondary metabolite production, without affecting the organism’s growth. Thus, a low resource constraint suggests that under varying nutrient bioavailability, growth remains relatively stable. In contrast, a high resource constraint indicates that only a small amount of the nutrient is available for nongrowth processes, making growth more sensitive to fluctuations in nutrient bioavailability. At its maximum ( 100% ), the resource constraint indicates that the given nutrient limits the organism’s growth, with all the bioavailable nutrients being used for growth.

**Fig. 2.**
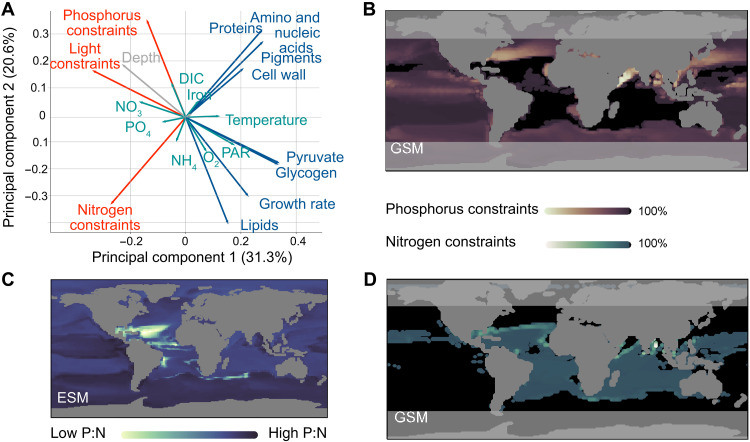
Simulation of *Prochlorococcus* **MED4** GSM across the global ocean. (**A**) Principal component analysis of over $10^{6}$ predictions in the global ocean, across depths and over an annual cycle. Physiological factors emphasized by GSMs are shown in blue for organismal composition and red for resource constraints, whereas environmental factors are shown in green, and those associated with biogeography are indicated in gray. (**B**) Distribution of phosphorus constraints based on orthophosphate uptake fluxes at the surface ocean in January. Lighter colors indicate no resource constraints on uptake. In contrast, higher resource constraints are depicted with darker colors until their maximum (indicated in black) when the nutrient is limiting per se. (**C**) Distribution of the phosphorus-to-nitrogen ratio estimated by NEMO-PISCES for picophytoplankton. (**D**) Distribution of nitrogen constraints based on ammonium uptake fluxes [the only source of nitrogen available to *Prochlorococcus* MED4 (*52*)] following the same color nomenclature as in (C).

On the basis of more than $10^{6}$ environmental conditions provided by NEMO-PISCES across space and time over an annual cycle in the global ocean, we estimated the growth and the resource constraints (for phosphorus, nitrogen, and light) of *Prochlorococcus* MED4 for each condition (Fig. 2A). We found that *Prochlorococcus* is not limited by light in the surface ocean but is regulated by nitrogen and phosphorus, which showed higher resource constraints. Specifically, the growth of *Prochlorococcus* MED4 is limited by phosphorus in the central Atlantic and the Indian oceans (Fig. 2B). This aligns with the quota estimation of NEMO-PISCES, which predicts a lower P:N ratio in these areas (Fig. 2C). Nitrogen-limited provinces are found in the Pacific, South, and North Atlantic oceans, which showed an antagonistic pattern between nitrogen and phosphorus resource constraints.

**Fig. 3.**
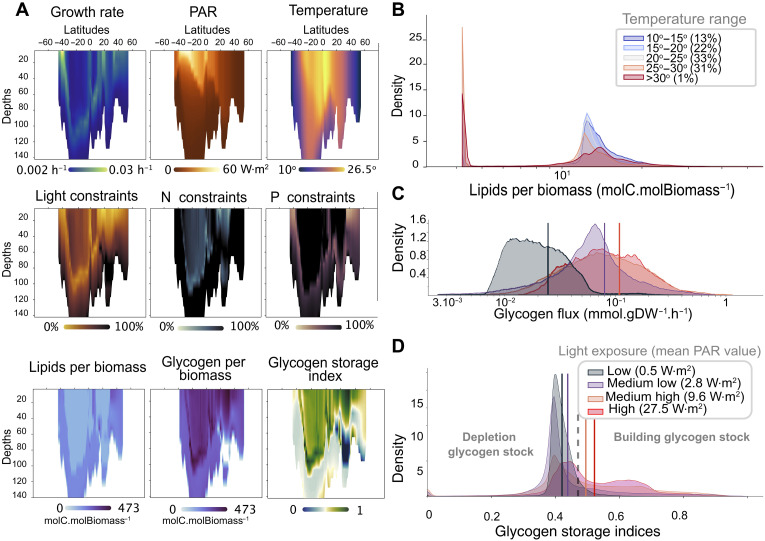
Investigation of *Prochlorococcus* **MED4** GSM fitness and acclimation strategies across the global ocean. (**A**) Description of *Prochlorococcus* MED4 metabolic behavior across the Atlantic Ocean transect (longitude of −24°). It describes growth rate according to light and temperature, associated nutrient constraints (light, nitrogen, and phosphorus), and acclimation consequences (glycogen and lipid productions per biomass, color code in log scale) resumed by the glycogen storage index (see Materials and Methods for details), with blue colors indicating the consumption of potential glycogen stock and green colors showing increased storage. (**B**) Distribution of lipid contribution to *Prochlorococcus* MED4 biomass over five distinct temperature ranges (see Materials and Methods for details). (**C**) Distribution of glycogen production satisfying predicted *Prochlorococcus* MED4 growth rates under four categories of gradual light exposures. (**D**) Distribution of glycogen storage indices computed with estimated *Prochlorococcus* MED4 growth rates under four similar categories of gradual light exposures. Indices between 0 and 0.46 indicate a gradual decrease in glycogen stocks to support growth. Above 0.46, indices are associated with full phototrophic growth with increased glycogen storage.

Along with resource constraints, the GSM estimated metabolite production pooled in quotas (e.g., proteins, amino acids, and cell wall) and metabolites involved in carbon storage (e.g., long-term storage for lipids and short-term storage for pyruvate and glycogen). Among the different types of carbon storage available to *Prochlorococcus* MED4, glycogen and lipid productions showed a weak correlation ( $R^{2}=0.5$ ) hinting at the organism’s different use of these metabolites. While the production of lipids and glycogen by *Prochlorococcus* MED4 is similar across the surface ocean, except in tropical Atlantic regions and the Bay of Bengal (fig. S10), they differ with depth and latitude (Fig. 3A). These findings highlight the complex response of *Prochlorococcus* MED4 to multifactorial limitations, without extensive model calibration but via the knowledge embedded in the metabolic network. These responses suggest acclimation strategies that require further investigation.

### GSM-based prediction of acclimation strategies of *Prochlorococcus* MED4 in the global ocean

Plankton are complex adaptive systems that could render them partially resilient to global changes (*28*). Understanding and incorporating their acclimation are central issues in ESM that GSMs can address. By investigating *Prochlorococcus* MED4 under each condition, we showed that provinces under high nitrogen constraints exhibit similar lipid and glycogen production patterns. However, when limited by phosphorus, *Prochlorococcus* MED4 showed more carbon storage in the form of glycogen than in lipids (Fig. 3A). It is explained by the fact that the lipid considered in the GSM is a pool of macromolecules, including phospholipids (see appendix S6.2 for details). Lipids are generally produced under extreme conditions for *Prochlorococcus* (i.e., at depth and latitudes around 40° and −40°; see Fig. 3A), far from the organism’s optimal growth conditions. By studying the distribution of *Prochlorococcus* MED4 lipid-to-biomass ratios across all possible environmental conditions, we found that it can increase threefold in cold waters (relative to <25° conditions; Fig. 3B), which is consistent with molecular evidence from other cyanobacteria (*31*).

When less constrained by light, *Prochlorococcus* MED4 growth is associated with the production of carbon compounds that are metabolically quicker to access, such as glycogen. High glycogen production is observed when carbon is stored minimally as lipids. Moreover, when both types of stocks are available, the observed difference in their production rates is due to the higher energy needed to produce lipids compared to glycogen (see appendix S6.2 for details). When grouped into categories of increasing light exposure, the mean value of glycogen production per category increased (Fig. 3C), linking this process to a photosynthetic behavior. In this respect, by investigating the inner machinery of *Prochlorococcus* MED4, we quantified the amount of carbon allocated to biomass or glycogen production. We defined the glycogen storage index as the normalized ratio of carbon allocated to glycogen production relative to the total amount of carbon fixed (see Materials and Methods for details). This index represents the organism’s capacity to store glycogen. A high index (i.e., 1) indicates a substantial use of carbon for glycogen storage while growing at maximal capacity. In contrast, a lower index (i.e., 0) suggests minimal glycogen production, with carbon directed primarily toward growth.

We interpret the lack of glycogen production as rerouting the carbon fixed, initially supposed to be stored as glycogen, into growth (see Materials and Methods for details). Below the mean glycogen storage index value (i.e., 0.46), *Prochlorococcus* MED4 combines photosynthesis and glycogen consumption (or lack of production) to ensure a higher growth rate, whereas, above this value, it shows a growth regime with glycogen storage or secretion. Our indices showed a natural tendency for the organism to undergo glycogen-deprived growth under low-light conditions (Fig. 3D). Conversely, MED4 exhibited both growth and glycogen overproduction in regions where phosphorus constrains growth rate, emphasizing the importance of estimating nitrogen and phosphorus constraints to assess growth regimes and uncover long-term versus short-term carbon storage strategies (i.e., lipids versus glycogen). These results confirm the importance of modeling planktonic metabolic flexibility (*22*) and the need to further design GSM for ocean studies, as already advocated by Casey *et al.* (*13*). This flexibility leads to highly complex and nonlinear relationships between the organism’s growth and its environment (Fig. 3A; pairwise linear correlation $R^{2}<0.2$ ), requiring deeper investigation, either through GSM and their metabolic niche (*21*) or through other modeling work that emphasizes biocomplexity (*9*). Patterns in resource constraints, which embed combinations of multiple limitations, provide a better explanation for *Prochlorococcus* MED4 growth. Acclimation to these constraints and environmental parameters, such as temperature, results in distinct carbon storage strategies, either in the form of lipids or as glycogen. Their respective production rates can thus reveal MED4’s prevalent acclimation strategy. However, glycogen and lipids are not the only carbon compounds represented in GSMs.

### Predicting hot spots of biotic production and metabolite diversity

**Fig. 4.**
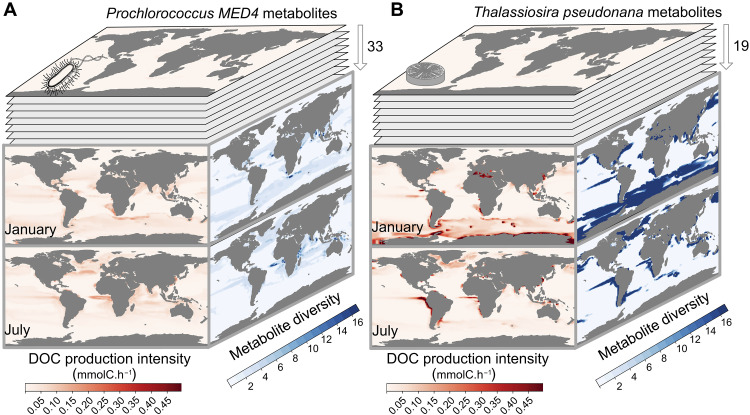
GSM-based predictions of DOC contributions from *Prochlorococcus* **MED4** and *T. pseudonana*. (**A**) *Prochlorococcus* MED4 produces 33 metabolites predicted to contribute to the DOC pool. Compilation of all compounds potentially contributing to DOC production is shown in red in January (top layer) and July (bottom layer). The diversity of produced metabolites during the same months is indicated in blue. (**B**) Same analysis as in (A) performed for the *T. pseudonana GSM*, which predicts *19* metabolites associated with the DOC pool. These analyses identify hot spots for DOC production intensity and associated metabolic diversity in January and July.

By assessing genome-scale knowledge, the use of GSMs through their metabolic niche is emerging as a valuable tool for investigating cellular composition (for detailed information and its application in designing improved trait models, refer to appendix S6.1) and diverse metabolite contents (*32*, *33*). As a case in point, specific metabolites play crucial roles in the labile DOC pool, a fundamental component of the ocean carbon cycle (*18*). They play a substantial role in bacterial and plankton growth (*34*). Unexpectedly, except in a few cases (*35*), current ESMs overlook this diverse range of metabolites when modeling DOC and instead represent a generic DOC pool. To address this shortcoming, we compiled a comprehensive summary of the DOC metabolite compounds listed in (*18*), specifically focusing on those produced by *T. pseudonana* and *Prochlorococcus* MED4. Our analysis revealed that *T. pseudonana* and *Prochlorococcus* MED4 GSMs produce 19 and 33 metabolites, respectively (see table S1 for detailed information). To estimate the contribution of each GSM to DOC production, we aggregated all the metabolite flux estimates (Fig. 4 and see Materials and Methods for details). In addition, we investigated the diversity and abundance of metabolites supporting DOC flux production considering seasonal variation across each GSM. Upwellings and fronts demonstrated higher DOC production and a wider array of secreted metabolites. GSM analysis shows opposite patterns in DOC production and metabolite diversity. Despite producing more diverse metabolites, *Prochlorococcus* MED4 shows restricted areas of high metabolite diversity within zones of high DOC production. On the contrary, *T. pseudonana* displays higher DOC production amidst wider areas of high diversity. By comparing both GSMs, *T. pseudonana* played a dominant role in both the magnitude of DOC production and the diversity of associated metabolites, with more expansive areas of DOC diversity aligning with the role of diatoms in shaping the biogeography of bacterial heterotrophs (*36*). Through a broader analysis of provinces, we distinguished regions driven predominantly by diatom influences from those more strongly affected by *Prochlorococcus*, reaffirming earlier findings (*37*). Notably, provinces exhibiting high metabolite diversity did not necessarily align with high DOC production, highlighting the importance of further investigation to understand the support for trophic interactions via metabolic cross-feeding between organisms (*38*) or to improve predictions concerning the relationship between functional diversity and ecosystem productivity (*34*, *39*).

## DISCUSSION

We estimate the growth rates of various organisms and validate those results against NEMO-PISCES estimates (pairwise correlations r>0.8 between modeled organisms and their corresponding tracers). Without added parameterization or modeling assumptions, we also emphasize the acclimation of *Prochlorococcus* to high-light exposure through glycogen storage and cold environments through lipid storage, supported by previous in vivo findings on cyanobacteria (*31*). We lastly explore the metabolic diversity accessible through GSMs by looking at DOC secretion of *Prochlorococcus* and *Thalassiosira* in terms of intensity and metabolite diversity. These DOC estimations result from ground-truthed metabolic behaviors such as growth rate (*24*), biogeography (*13*), and acclimation strategies (*22*). They call for further in situ validation via metaproteome studies (*40*) and better calibration of GSMs to DOC secretions. However, they already underscore the potential of integrating genomic knowledge into ESMs for predicting DOC or other biotic factors essential for climate prediction (*19*).

Our modeling paradigm is one step toward embedding molecular biocomplexity into ESM, overcoming some limitations in current models while introducing new challenges. Rather than claiming to be the only solution, we hope that our work will encourage many more attempts at enhancing the representation of biology within ESMs (*19*). Our framework can be further extended to incorporate more environmental omic data into ESMs, for instance, using GSMs constructed from metagenome-assembled genomes (*41*) or proteomics-based data (*40*, *42*, *43*). Moreover, because of seminal links between evolutionary theory and metabolic network (*44*), our approach paves the way for future integration of adaptation in climate models (*28*, *45*). The remaining challenges for developing a fully integrated genome-enabled ESM are clear. First, growth metrics from GSMs must integrate seamlessly into ESM tracers, translating growth rates into organismal abundance. Second, GSM uptake metrics should better reflect actual substrate use, not just bioavailability. Resolving these challenges will enable ESM’s progress, such as adding GSM-driven tracers such as DOC metabolites (*18*) or other micronutrients essential for trophic interactions (*46*), and modeling more organisms. Existing GSMs cover bacteria, diatoms, and nanophytoplankton, but there is potential for developing ecotype-specific models (*13*) or iconic species such as calanoid from different trophic levels.

Last, genome scale–enabled ESMs have the potential to advance our understanding of the intricacy of Earth’s microbial ecosystems and global ocean biogeochemical processes (*47*). Consequently, computational modeling could better connect intergovernmental panels’ conclusions addressing climate change and biodiversity loss.

## MATERIALS AND METHODS

### Genome-scale model

A GSM is stated as a set of linear constraints, representing the quasi–steady state assumption, and the thermodynamic constraints

$$\left\{ \begin{array}{cc} \;\text{Sv}=0 & \\ \;l_{b}\leq v\leq u_{b}\; & \end{array}\right.$$ (1)

The matrix $S\in {\mathbb{R}}^{n,m}$ abstracts the metabolic network of *n* metabolites and *m* reactions, the vector $v\in {\mathbb{R}}^{m}$ represents the fluxes that go through each network reaction, and $l_{b},u_{b}\in {\mathbb{R}}^{m}$ are the lower and upper bounds of v . To represent the organism’s growth rate, metabolic models include a biomass reaction, which specifies the metabolic requirements for organismal growth. It is included in S and cannot have a negative flux. Given the stated problem in Eq. 1, one can calculate and extract the flux for each network reaction, including the biomass reaction. A solution of Eq. 1 is one of the feasible physiological states of the system. In this state, one can estimate the organism’s growth rate as the flux through the biomass reaction. Further details on the metabolic framework and GSM are provided in appendix S1.

### Metabolic niche projection

We call solution space F the convex hypervolume composed of v satisfying Eq. 1. F is defined in a space where each dimension represents the flux through a reaction. Investigation of this space is subject to numerous techniques in the context of metabolic engineering [see Price *et al.* (*48*) for review]. However, using F is not well suited in biogeochemical models because of its size and complexity. In most cases, one cannot describe the entire shape of F, as its complexity grows exponentially with the number of reactions of the GSM. In addition, biogeochemical models describe the distribution of a few nutrients compared to the number of metabolites in a GSM. It means that most of the reactions and underlying mechanisms of the metabolic network can be abstracted in favor of a numerical tool, linking the exchange reactions relative to nutrients available in biogeochemical models to the biomass reaction and enabling the estimation of growth rates. In mathematical terms, it implies projecting F onto a smaller space composed of the sole reactions of interest (i.e., reactions representing a parameter in the biogeochemical model and the biomass reaction). This projection, called the metabolic niche, is computed through multiobjective linear programming (*49*). Decomposing the flux vector $v=\left(\begin{array}{c} x\\ y \end{array}\right)$ , where x is a flux vector composed of the reactions of interest (i.e., the exchange reactions concerning the nutrient and light absorption) and y is a flux vector composed of all the other reactions. The projection of F is equivalent to solving the following problem

$$\left\{ \begin{array}{cc} \text{min}\left(\begin{array}{c} I_{p}\\ -1_{p}^{T} \end{array}\right)x & \\ \text{subject to}\;\left(\begin{array}{c} x\\ y \end{array}\right)\in F\; & \end{array}\right.$$ (2)

where $I_{p}$ is the identity matrix in ${\mathbb{R}}^{p\times p}$ , $1_{p}$ is the column vector composed of ones in ${\mathbb{R}}^{p}$ , and $1_{p}^{T}$ is its transposition. The solution of Eq. 2 without its last component gives a set of vertices describing the convex hypervolume N . Applied to several organisms, the investigation of the metabolic niche hypervolume has shown ecological properties (*21*).

### Description of the biogeochemical model

Our framework is interoperable with the marine biogeochemical model PISCES. PISCES is the biogeochemical component of the NEMO modeling platform. This study uses the quota version of PISCES (*20*). Three phytoplankton groups are explicitly modeled (picophytoplankton, nanophytoplankton, and diatoms) whose growth rates are limited by iron, phosphate, nitrate, ammonium, and silicate availability. Two zooplankton groups (micro- and mesozooplankton) are represented. PISCES also models dissolved oxygen, particulate and dissolved organic matter, and calcite. The uptake of nutrients and phytoplankton growth rates are modeled using the quota formalism. Metabolic rates increase with temperature according to the commonly used Eppley parameterization (*50*). On the basis of the environmental conditions and the biotic interactions between the different plankton groups, NEMO-PISCES estimates the growth rate for each plankton group using partial differential equations.

### The metabolic niche in NEMO-PISCES

Because of the metabolic niche projection, we can compute the growth rate of an organism based on the environmental conditions dictated by NEMO-PISCES. Thus, biology will be handled by omic-derived knowledge of metabolic models, while NEMO-PISCES will compute the nutrient bioavailability. NEMO-PISCES’s simulations provide essential nutrients for algal growth, including iron, nitrate, ammonium, phosphate, silicate (used only by diatoms), and the quantity of carbon fixed by photosynthesis. These nutrient fluxes are incorporated into the distinct metabolic niches represented by GSMs of these generic phytoplankton. While *Prochlorococcus* MED4 cannot assimilate nitrate, it uses ammonium, iron, and phosphate. NEMO-PISCES inputs constrain the exchange reactions of the previous metabolite and the 3-phospho-d-glycerate carboxylase reaction for the quantity of carbon fixed. However, our calculations did not incorporate iron (see appendix S6.3). In the context of generic diatom modeling (*T. pseudonana* and *P. tricornutum*), iron was not included in their GSMs (*22*, *24*). The equivalent reaction for the carbon-fixed quantity is the carboxylation of ribulose-1,5-bisphosphate, called RUBISC_h in both models. Adding to these reactions, the biomass reaction, which estimates the growth rate, together, they form the reactions of interest that will compose each organism’s metabolic niche.

GSMs predict growth rates outside of their thermal range (Fig. 1E, gray areas for *Prochlorococcus* MED4), as the modeling paradigm does not incorporate the thermal tolerance of the modeled organisms, indicating that the sole metabolism does not include processes involved in the thermal tolerance. This absence of thermal effect in the GSM does not change the overall results, as the temperature is modeled in NEMO-PISCES (*50*) and affects the uptake fluxes of nutrients.

### Growth rate extraction from the metabolic niche

The metabolic niche describes the ability of an organism to survive and grow considering its genome-scale metabolic description under particular environmental conditions. Conceptually, the metabolic niche algorithm estimates the flux range of a reaction of interest under given constraints on other reactions. Here, the constraints, which represent the environment, relate to nutrient uptake as described by the ESM. The reaction of interest is the biomass reaction. That is, we used the metabolic niche algorithm to determine the maximal growth allowed by the GSM under the environmental conditions computed by NEMO-PISCES.

Formally, considering a vector of uptake fluxes given by the biogeochemical model $x_{\text{envb}}$ , we construct $x=\left(\begin{array}{c} x_{\text{envb}}\\ x_{\text{bio}} \end{array}\right)$ , where $x_{\text{bio}}\in {\mathbb{R}}^{+}$ is the flux through the biomass reaction. Then, we need to look for the maximal $x_{\text{bio}}$ that satisfies x∈N for a particular $x_{\text{envb}}$

$$\left\{ \begin{array}{cc} \text{max}\;x_{\text{bio}} & \\ x=\left(\begin{array}{c} x_{\text{envb}}\\ x_{\text{bio}} \end{array}\right)\in N & \end{array}\right.$$ (3)

where N is the metabolic niche described above. There are two cases of this problem—either a solution exists, and we can solve the problem and output the solution, or there is no solution, meaning that the environmental condition does not belong to the niche. In this case, the organism cannot grow, and the growth rate should be fixed to 0. However, instead of regarding $x_{\text{envb}}$ as a fixed nutrient uptake, we can view it as the bioavailability of nutrients. In this context, nutrient bioavailability does not represent the actual uptake of the organism; rather, it represents the upper limit of nutrient uptake. That is, the organism cannot take up more nutrients than what is available.

### Nutrient bioavailability from the NEMO-PISCES biogeochemical model

The organism is not necessarily using all the resources of its environment. The metabolic network should handle the quantity of nutrients it consumes. If we denote $x_{\text{envb}}$ as the quantity of bioavailable nutrients, and $x_{\text{env}}$ as the actual nutrient fluxes used by the model, then we need to assure that $x_{\text{env}}\leq x_{\text{envb}}$ . We depict this as an additional constraint on the uptake fluxes, which changes the formulation of Eq. 3. Thus, we seek the maximum of $x_{\text{bio}}$ that satisfies $x_{\text{env}}\leq x_{\text{envb}}$ and $\left(\begin{array}{c} x_{\text{env}}\\ x_{\text{bio}} \end{array}\right)\in N$ . The formulation is, therefore

$$\left\{ \begin{array}{cc} \text{max}\;x_{\text{bio}} & \\ \;x_{\text{env}}\leq x_{\text{envb}} & \\ x=\left(\begin{array}{c} x_{\text{env}}\\ x_{\text{bio}} \end{array}\right)\in N & \end{array}\right.$$ (4)

where $x_{\text{env}}$ is the actual uptake fluxes used by the GSM and constrained by $x_{\text{envb}}$ the uptake fluxes computed by NEMO-PISCES. This formulation assures a solution to the problem.

### Auxiliary flux computation

Not only can the metabolic niche produce growth rate estimates, but it can also estimate fluxes through any reaction in the GSM. Using bi-level optimization, one can compute the metabolic niche with an additional dimension and analyze flux variability along this dimension.

Conceptually, the metabolic niche estimates bounds for an unknown flux under a set of constraints. The two-step bi-level optimization process works as follows:

1) Apply the niche algorithm with the biomass reaction as the unknown and nutrient bioavailability as the constraints. This step outputs the maximum flux value for the biomass reaction.

2) Fix the biomass reaction flux at its maximum value in the model. Then, reapply the niche algorithm with the auxiliary reaction as the unknown and nutrient bioavailability as the constraints.

Taking the former formalism for x , we can write $x=\left(\begin{array}{c} x_{\text{env}}\\ x_{\text{bio}}\\ x_{\text{aux}} \end{array}\right)$ , where $x_{\text{aux}}\in \mathbb{R}$ is the flux through another reaction of the network, say, the flux through the exchange reaction of DMSP, or the modeling reaction producing the organism pigment. With the previous method, we can determine the maximal $x_{\text{bio}}$ with respect to $x_{\text{env}}$ , which gives us $x_{\text{known}}=\left(\begin{array}{c} x_{\text{env}}\\ x_{\text{bio}} \end{array}\right)$ . Applying the same computation on $x=\left(\begin{array}{c} x_{\text{known}}\\ x_{\text{aux}} \end{array}\right)$ gives us a range of flux under the environmental condition defined by PISCES and the assumption that the organism is maximizing its growth rate. Rewriting Eq. 4, with $x_{\text{aux}}$ and computing not only the maximum value but also its minimum, we have the following problem

$$\left\{ \begin{array}{cc} \text{min}/\text{max}\;x_{\text{aux}} & \\ \;w.r.t\;\text{max}\;x_{\text{bio}} & \\ w.r.t\;x=\left(\begin{array}{c} x_{\text{env}}\\ x_{\text{bio}}\\ x_{\text{aux}} \end{array}\right)\in N & \end{array}\right.$$ (5)

Once solved, it gives us the flux range of the reaction of interest. This method can be applied to internal or exchange reactions.

While the biomass reaction of a GSM has a fixed stoichiometry, we can add an exchange reaction for the components of interest to enable virtual quantification of their production, with an additional constraint: The model is restricted to producing the component, not uptaking it (see appendix S1.2 for details). This bi-level optimization estimates the (over)production of metabolites with respect to the organism’s growth without altering the original metabolic behavior of the model. Adenosine 5′-triphosphate or the reduced form of nicotinamide adenine dinucleotide phosphate costs associated with the production of those components are not taken into account here. However, when these costs become available, the model’s flexibility will allow for their incorporation, enabling more accurate estimations.

To obtain our results, we compute the auxiliary fluxes sequentially, handling each metabolite independently. Further work is required to model the simultaneous secretion of multiple metabolites.

### Variable biomass composition

While the biomass composition is fixed in GSMs, we can estimate variable ratios of metabolites per unit of biomass. Consider metabolite *M*, whose fixed ratio is determined by the biomass equation of the model and denoted *a*. Thus, $\frac{M}{B}=a$ , where *M* and *B* represent the amount of metabolite *M* and biomass, respectively, in the modeled organism. If an exchange reaction for *M* exists, then we can virtually include the flux through this reaction in the biomass composition. We denote $x_{M}$ and $x_{\text{bio}}$ as the fluxes through the exchange reaction of metabolite *M* and the biomass reaction, respectively. The flux of metabolite *M* produced by the model is then $x_{M}+{\mathrm{ax}}_{\text{bio}}$ . Dividing this flux by $x_{\text{bio}}$ yields the quantity of M per unit of biomass. To enable comparison between different ratios, we transform them into carbon equivalents by multiplying by their respective carbon stoichiometry coefficient. In the case of *Prochlorococcus* MED4 GSM, for lipids, the carbon-equivalent ratio is calculated as $r_{\text{lipid}}=33\times \frac{x_{\text{lipid}}+0.128x_{\text{bio}}}{x_{\text{bio}}}$ , as, in the model, the lipid macrometabolite has a stoichiometric coefficient of 33 for its carbon content and a stoichiometric coefficient of 0.128 in the biomass equation. For glycogen, we have $r_{\text{glyc}}=7\times \frac{x_{\text{glyc}}}{x_{\text{bio}}}$ , as the glycogen does not participate in the biomass reaction since Ofaim *et al.* (*22*) and has a stoichiometric coefficient of 7 for its carbon content.

### Carbon cycle hot spots

Carbon hot spots were identified using the auxiliary flux computation as described above. Metabolites used for the computation are the same as those described in (*18*) and found in the GSM of *Thalassiosira* or *Prochlorococcus* with an exchange reaction. Each flux was scaled with the carbon content of the corresponding metabolites (see appendix S1.4 for details), and its unit is thus millimoles of carbon per gram of dry weight per hour. To generate the intensity of DOC production at each grid point, we multiply the highest flux value by the abundance of the organism, giving us a flux in millimoles of carbon per hour. The abundance used was the one computed by NEMO-PISCES, that is, the entire diatom abundance for *Thalassiosira* and the entire picophytoplankton abundance for *Prochlorococcus*. We included a metabolite in the diversity score if its production was above 5% of the maximum flux computed among all other metabolites.

### Resource constraint estimate

Our results show that when an organism is under the limitation of one nutrient, the others are in excess. Briefly, the resource constraint represents the quantity of nutrients that can be allocated to other metabolic pathways than those linked to biomass production. Our resource constraint definition is proportional to the amount of nutrient that is in excess. We can write the resource constraint on the nutrient *n* as ${\mathrm{RC}}_{n}∼-\;\delta n$ , where δn is the quantity of the nutrient *n* that the organism can use for something other than its growth. Hence, a high resource constraint means that the nutrient almost limits the production of biomass ( δn∼0 ). In contrast, a low resource constraint means more nutrient *n* can be used for other products such as energy storage or other organic compound secretion.

Formally, we first consider the vector d between $x=\left(\begin{array}{c} x_{\text{env}}\\ x_{\text{bio}} \end{array}\right)$ and $x_{b}=\left(\begin{array}{c} x_{\text{envb}}\\ x_{\text{bio}} \end{array}\right)$ , where $x_{\text{bio}}$ is the solution of Eq. 4. $x_{\text{env}}$ is the quantity of nutrients used by the model to produce $x_{\text{bio}}$ of growth, whereas $x_{\text{envb}}$ is the bioavailability of nutrients. Each component of the computed vector is a quantity of nutrient not used by the model. As this vector is defined for each environmental condition (we denote env∈E an environmental condition and E the ensemble of environmental condition), we then normalize the distribution of each component *n* corresponding to one nutrient, to get a value between 0 and  100%

$${\mathrm{RC}}_{n}\left(\text{env}\right)=\frac{d_{n}-{\underset{\text{env}\in E}{\text{min}}}_{}\left(d_{n}\right)}{\underset{\text{env}\in E}{{\text{max}}_{}}\left(d_{n}\right)-{\underset{\text{env}\in E}{\text{min}}}_{}\left(d_{n}\right)}$$ (6)

### Glycogen storage index

Our glycogen storage index is based on the production of glycogen and the quantity of carbon fixed by the organism. Formally, we write $r_{\text{stor}}=\frac{\text{glycogen produced}}{\text{carbon fixed}}$ as the glycogen storage ratio that we normalize to give our index. “Glycogen produced” is the flux of glycogen for a given condition and a growth rate. “Carbon fixed” is the quantity of carbon fixed, provided by NEMO-PISCES. This ratio represents the percentage of carbon fixed used to produce glycogen. That is, it looks at the resource allocation of the organism. The storage or secretion of glycogen can be used by *Prochlorococcus* MED4 to adapt to different environmental conditions. From the mean value of the index, we distinguish two types of growth. Suppose that the ratio is higher than its mean value. In that case, the organism is already growing at its full potential considering its environment and can store the excess carbon fixed into glycogen. On the other hand, an index below the mean indicates that more carbon fixed is used for the biomass, and the lack of glycogen produced can be seen as consumption: The final flux—the difference between the mean index and the current index—is proportional to the quantity of glycogen needed by the organism to grow in a particular environment. Formally, it implies $v_{\text{glycogen}}={\bar{v}}_{\text{glycogen}}-{\tilde{v}}_{\text{glycogen}}$ , where $v_{\text{glycogen}}$ is the actual glycogen production, ${\bar{v}}_{\text{glycogen}}$ is the glycogen production associated with the mean value of the glycogen storage index, and ${\tilde{v}}_{\text{glycogen}}$ is what is consumed by the organism to sustain its growth. This modeling artifact does not take into account the energy needed to store or consume the artificially created glycogen ( $v-\tilde{v}$).

### Limitations of the model

Our framework, similar to any modeling approach, has certain limitations (see appendix S4.2 for more details). In our model, light is represented by the quantity of carbon fixed, which organisms can use for growth or other metabolic processes. However, organisms cannot choose to forgo the utilization of light; instead, they adapt their composition to absorb varying amounts of photons through photoadaptation and photoinhibition mechanisms. These mechanisms, however, are not represented in the GSM, which explains the observed results related to pigment production (see appendix S6). Furthermore, the quantity of carbon fixed relies on a parameter α (photosynthetic efficiency), which assumes uniformity across all organisms, despite experimental evidence indicating variations in these parameters (*51*).

The biomass reaction approximates the growth rate of the GSM. However, this reaction is constructed on the basis of laboratory experiments that may not fully capture in situ environmental conditions. For example, while iron is present in the *Prochlorococcus* MED4 GSM, its utilization is not possible because of stoichiometric differences compared to NEMO-PISCES (see appendix S6.3). As the internal processes represented in GSM are abstracted by the niche algorithm, variations in kinetic parameters that enable organisms to acclimate to different environments are only accounted for through the variation of input fluxes computed by NEMO-PISCES.

Last, all simulations are conducted offline. Initially, NEMO-PISCES is executed, and subsequently, growth is diagnosed using the GSMs. Further work is necessary to fully integrate the GSMs into NEMO-PISCES.

## Supplementary Materials

### This PDF file includes:

Appendices S1 to S7 Figs. S1 to S14 Tables S1 to S3 References
