# Supplementary material for: Unveiling the link between phytoplankton molecular physiology and biogeochemical cycling via genome-scale modeling
Source: Sci Adv. 2025 Jun 4;11(23):eadq3593. doi: 10.1126/sciadv.adq3593 (PMC12136029; doi:10.1126/sciadv.adq3593)
Supplement: Supplementary file 1 — Appendices S1 to S7 Figs. S1 to S14 Tables S1 to S3 References [file sciadv.adq3593_sm.pdf]

Science Advances
AAAS

Supplementary Materials for

Unveiling the link between phytoplankton molecular physiology and biogeochemical cycling via genome-scale modeling

Antoine Régimbeau *et al.*

Corresponding author: Antoine Régimbeau, antoine.regimbeau@hawaii.edu;
Damien Eveillard, damien.eveillard@univ-nantes.fr

Sci. Adv. **11**, eadq3593 (2025)
DOI: 10.1126/sciadv.adq3593

This PDF file includes:

Appendices S1 to S7
Figs. S1 to S14
Tables S1 to S3
References

Appendix 1: Genome-scale model formalism and its manipulation

1.1 Genome scale model

From the genome or the proteome of an organism, one can associate reactions representing the metabolic abilities of an organism (*53*). Some of those reactions make reactants for others, with this set of reactions denoted as a metabolic network. From this network, one can build a Genome-Scale Model (GSM) that focuses on the flux carried by each reaction under some constraints. We differentiate two types of reaction in a GSM: the internal reactions involving metabolites that represent the organism machinery and the exchange reactions involving external and internal metabolites that describe the exchanges between the organism and its environment. For a reaction R_i, we define the stoichiometric coefficient for each internal metabolite j by:

$$s_{ij} = \begin{cases} -\alpha & \text{if } R_j \text{ consumes } \alpha \text{ molecules of } M_i \\ & \quad \text{in its forward direction,} \\ \alpha & \text{if } R_j \text{ produces } \alpha \text{ molecules of } M_i \\ & \quad \text{in its forward direction,} \\ 0 & \text{if } R_j \text{ neither produces nor consumes } M_i. \end{cases}$$

By convention, exchange reactions are written as:

$$M_i \longleftrightarrow M_i ex \quad ,$$

where $M_i ex$ is the external metabolite M_i. Hence, the forward reaction means that the system adds M_i into the environment, whereas a reverse reaction means that the system consumes M_i from the environment. Consider a metabolic network of n reactions and m internal metabolites. According to kinetic theory, the change over time of the concentration of the metabolite i is given by the mass balance equation:

$$\frac{d[M_i]}{dt} = s_{i1}v_1 + s_{i2}v_2 + \ldots + s_{in}v_n = \sum_{j=1\ldots n} s_{ij}v_j \quad , \tag{S1}$$

where $v_j \in \mathbb{R}$ is the reaction rate or flux associated with reaction R_j and s_{ij} are the stoichiometric coefficient described above. Fluxes here are expressed as a mole of product formed (or mole of reactant consumed) per gram of dry weight of the considered organism per hour, i.e., mol.gDW^{-1}.h^{-1}. We can write the above equation for all internal metabolites expressed in vector notation as:

$$\frac{d\mathbf{M}}{dt} = \mathbf{Sv} \quad , \tag{S2}$$

where $\mathbf{S} \in \mathbb{R}^{(n,m)}$ is called the stoichiometric matrix of the network, $\mathbf{v} \in \mathbb{R}^n$ the flux vector representing the flux carried by each reaction, and $\mathbf{M} \in \mathbb{R}^{+n}$ the vector composed of each metabolite concentration $[M_i]$. Based on the principle that environmental changes are very slow compared to metabolic adjustments (*54*), one can assume the system at quasi-steady-states, linearising the above equations to:

$$\mathbf{Sv} = 0 \quad . \tag{S3}$$

Kinetic parametrization of reactions is not well suited to our framework as it is computationally very demanding and needs extensive kinetic data to estimate enzymatic activities (*55*). Instead, GSM uses bounds, representing the thermodynamic feasibility of the reaction. A reaction cannot have an infinite flux. Thus each v_i are constraint as follow:

$$lb_i \leq v_i \leq ub_i \quad , \tag{S4}$$

where ub_i represents the upper bound of the flux, meaning the highest rate of the direct reaction, and lb_i represents the lower bound of the flux, i.e., the highest rate of the reverse reaction. Moreover, the irreversibility of the reaction can be translated into thermodynamic constraint. For instance, a reaction known to be direct and irreversible will have a positive flux:

$$0 \leq v_i \leq ub_i \quad . \tag{S5}$$

Hence the GSM is stated as a set of linear constraints:

$$\begin{cases} \mathbf{Sv} = \mathbf{0} \\ \mathbf{l_b} \leq \mathbf{v} \leq \mathbf{u_b} \quad , \end{cases} \tag{S6}$$

To represent the growth rate of the organism, metabolic models include a biomass reaction that describes the metabolic requirement for an organism to grow. It is included in the matrix **S** and cannot have a negative flux. Given the stated problem, one can calculate with a dedicated solver and extract the flux for each network reaction, including the biomass reaction. The solution is one of the feasible physiological states of the system. In this state, one can estimate the organism's growth rate as the flux through the biomass reaction.

1.2 Growth rate and biomass components

Most models are provided with a biomass objective function in the form of a synthetic reaction. This reaction encloses the metabolic need for the organism to grow. In the case of *Prochloroccocus MED4*, the biomass reaction requires several metabolites (see fig. S10) (*22*). All those compounds have a fixed stoichiometry in the model and allow biomass production, i.e., the organism's growth.

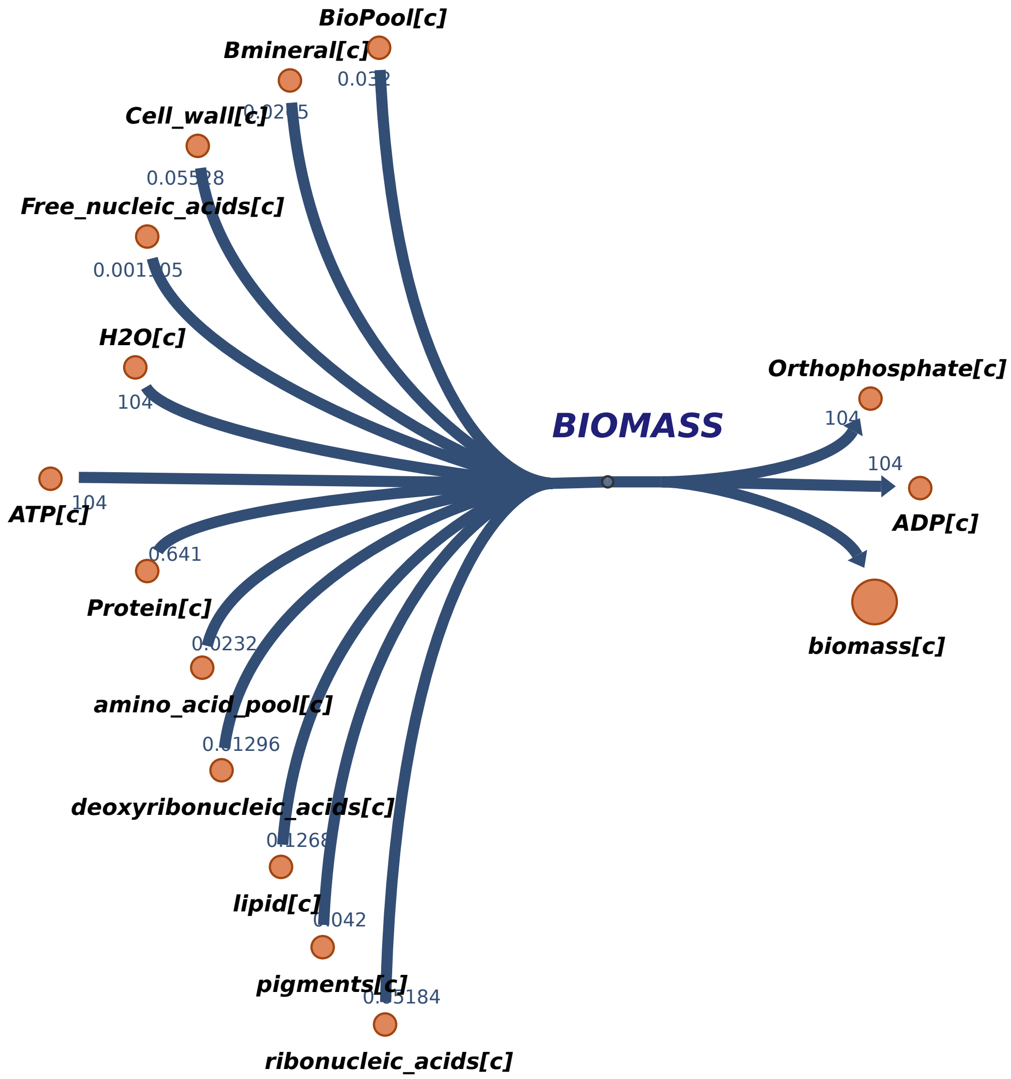

Figure S1: Biomass reaction of *Prochloroccocus MED4* in the model iSO595 (produced with Escher)

Most of the fictive metabolites have the only purpose of meeting the metabolite requirement for biomass production. Hence, they are not consumed elsewhere.

To compute an overproduction of those components, we created exchanges reactions allowing the organism to produce them without necessarily using them for biomass production. Those exchange reactions were restricted to production only (lower bound of 0). Hence, these changes in the metabolic network do not change the growth computed through our formalism but enables us to compute the overproduction of these metabolites, allowing us to investigate the organism's physiology and step away from the fixed stoichiometry imposed by the metabolic framework.

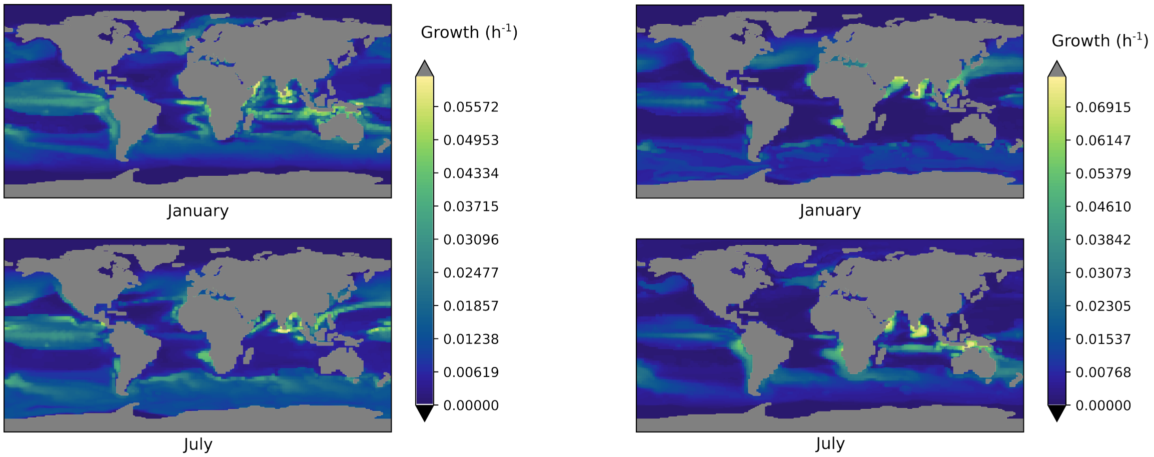

(a) *Prochloroccocus MED4* **(b)** *Thalassiosira pseudonana*

Figure S2: Growth of *Prochloroccocus MED4* and *Thalassiosira pseudonana*

Further investigation is needed to fully allow the GSM to control its biomass composition.

1.3 Resource constraint interpretations

The resource constraint can be linked to a metabolic stress measure. For instance, in a strongly constrained environment (resource constraint near 100%), the growth is assumably limited by the considered nutrient.

Moreover, the resource constraint can be used with a growth allocation metric to further study the modeled organism. The growth allocation for nutrient i would be defined as the ratio of the actual uptake flux of nutrient over the bioavailability of the nutrient $ga_i = \frac{x_{env,i}}{x_{envb,i}}$, where $\mathbf{x}_{envb} = \begin{pmatrix} \mathbf{x}_{env} \\ x_{bio} \end{pmatrix}$ and $\mathbf{x}_{env} = \begin{pmatrix} \mathbf{x}_{envb} \\ x_{bio} \end{pmatrix}$ as defined in Material & Methods. Hence, a growth allocation of 1 is equivalent to a constraint of 100%, meaning that all the available nutrients are used to produce growth. Lower values of growth allocation can be compared to the associated resource constraint to see the percentage of used nutrients for growth (growth allocation) and the available nutrient for auxiliary metabolism (resource constraint).

1.4 DOC metabolites

Table S1: Lists of metabolites that contribute to Dissolved Organic Carbon for each GSM, with their stoichiometric coefficient for carbon.

Prochloroccocus MED4	*Thalassiosira pseudonana*
L-Arginine (6), L-Asparagine (4), L-Aspartate (4), L-Glutamate (5), L-Glutamine (5), Glycine (2), L-Histidine (6), L-Isoleucine (6), L-Leucine (6), L-Lysine (6), L-Methionine (5), L-Phenylalanine (9), L-Proline (5), L-Serine (3), L-Threonine (4), L-Tryptophan (1), L-Tyrosine (9), L-Valine (5), D-Glucose (6), Acetate (2), Citrate (6), Glutathione (10), Pantothenate (9), Succinate (4), Thymidine (10), Xanthosine (10), 4-Aminobenzoate (7), 5-Methylthioadenosine (11), Adenine (5), Guanine (5), Guanosine (10), Putrescine (4), Spermidine (7), 4-Hydroxybenzoate (7), S-Malate (4)	Folate (19), N-acetyltaurine (4), Acetate (2), Choline (5), Uracil (4), Xanthine (5), L-Glutamate (5), L-Aspartate (4), L-Isoleucine (6), L-Leucine (6), L-Valine (5), L-Asparagine (4), L-Alanine (3), L-Glutamine (5), L-Histidine (6), L-Serine (3), L-Threonine (4), Glycine (2), L-Proline (5), Adenosine monophosphate (10), 3-(dimethylsulfonio)propanoate (5)

Appendix 2: Connection between GSM and NEMO-PISCES

2.1 From concentration to fluxes

Uptake of PO4 :

$$U_{PO4} = U_P^{max} b^T \left(\frac{Q_N^{min}}{Q_N}\right) L_{lim}^{Fe} \frac{(1 - \frac{Q_P}{Q_P^{max}})^2}{0.05^2 + (1 - \frac{Q_P}{Q_P^{max}})^2} L_{PO4} \tag{S7}$$

Uptake of NO3 and NH4:

$$U_{NO3} = U_N^{max} b^T \left(\frac{Q_N^{min}}{Q_N}\right) \frac{(1 - \frac{Q_N}{Q_N^{max}})^2}{0.05^2 + (1 - \frac{Q_N}{Q_N^{max}})^2} \times \min\left(L_{lim}^{Fe}, \frac{Q_P - Q_P^{min}}{Q_P^{max} - Q_P^{min}}\right) L_{NO3}(1 - L_{NH4}) \tag{S8}$$

$$U_{NH4} = U_N^{max} b^T \left(\frac{Q_N^{min}}{Q_N}\right) \frac{(1 - \frac{Q_N}{Q_N^{max}})^2}{0.05^2 + (1 - \frac{Q_N}{Q_N^{max}})^2} \times \min\left(L_{lim}^{Fe}, \frac{Q_P - Q_P^{min}}{Q_P^{max} - Q_P^{min}}\right) L_{NH4} \tag{S9}$$

where:

$$L_{NO3} = \frac{NO3}{NO3 + K_{NH4}} \tag{S10}$$

$$L_{NH4} = \frac{NH4}{NH4 + K_{NO3}} \tag{S11}$$

$$L_{lim}^{Fe} = \min(1, \frac{Q_{Fe} - Q_{Fe}^{min}}{Q_{Fe}^{opt}}) \tag{S12}$$

Uptake of iron :

$$U_{Fe} = U_{Fe}^{max} b^T \left(\frac{Q_N^{min}}{Q_N}\right) \frac{(1 - \frac{Q_{Fe}}{Q_{Fe}^{max}})^2}{0.05^2 + (1 - \frac{Q_{Fe}}{Q_{Fe}^{max}})^2} \times (1 + 0.8 \frac{L_{NO3}}{L_{NO3} + L_{NH4}} \times \frac{K_{Fe}}{Fe + K_{Fe}}) \times L_{Fe} \tag{S13}$$

All maximum nutrient uptake rates in the above equations are computed as:

$$U_i^{max} = V_i^{max}(1 - \xi \frac{Chl}{C}) \quad \text{(S14)}$$

In fact, in the equations above, limitation of the uptake rate by the availability in nutrients follow the optimal uptake formalism:

$$\begin{aligned} f_A &= \frac{1}{1 + \sqrt{\frac{A}{K_A}}} \\ L_A &= (1 - f_A)\frac{A}{\frac{f_A}{1-f_A}K_A + A} \end{aligned} \quad \text{(S15)}$$

Uptake of Si :

$$U_{Si} = V_C^{max} b^T Q_{Si}^o F_{Si}^{0.75} R_{Si} \frac{(1 - \frac{Q_{Si}}{Q_{Si}^{max}})^4}{0.1^4 + (1 - \frac{Q_{Si}}{Q_{Si}^{max}})^4} \frac{Si}{K_{Si} + Si} \quad \text{(S16)}$$

Where

$$\begin{aligned} Q_{Si}^{max} &= R_{Si} \times Q_{Si}^M \\ R_{Si} &= 1 + \frac{Si^3}{K_S^3 + Si^3} \quad \text{If latitude} < -30 \\ R_{Si} &= 1 \quad \text{otherwise} \end{aligned} \quad \text{(S17)}$$

and where F_{Si}^{lim} equals 1 when Si availability is limiting primary production (Carbon production). Otherwise, it equals

$$F_{Si} = \frac{U_C}{V_C^{max} b^T} \quad \text{(S18)}$$

Primary production (carbon production):

$$U_C = V_C^{max} b^T \min\left(L_N^{lim}, L_{Fe}^{lim}, L_{Si}^{lim}\right) \times \left(1 - \exp(-\frac{\alpha \theta_{Chl} I}{V_C^{max} b^T \min(L_N^{lim}, L_{Fe}^{lim}, L_{Si}^{lim})})\right) \times L_D^{lim} \quad \text{(S19)}$$

Where I is the light intensity and θ_{Chl} is the chlorophyll to carbon ratio of the cell. And Lim_D is the impact of light duration that depends on $strn$ the day length in hours:

$$\begin{aligned} L_D^{lim} &= 1 - exp(-0.26 * L_D) \\ L_D &= \max(1, strn) \end{aligned} \tag{S20}$$

$$\begin{aligned} L_N^{lim} &= \left(\frac{Q_N - Q_N^{min}}{Q_N^{max} - Q_N^{min}} \right) \frac{Q_{max}^N}{Q_N} \\ L_{Fe}^{lim} &= \min(1, \frac{Q_{Fe} - Q_{Fe}^{min}}{Q_{Fe}^{opt}}) \\ L_{Si}^{lim} &= \frac{Si}{Si + K_{Si}^{lim}} \end{aligned} \tag{S21}$$

The minimum iron quota is a variable function. It is described in Aumont et al. (2015) (*4*), see equation 20 of that paper. The half saturation constant of Si K_{Si}^{lim} is also computed as in Aumont et al. (2015), see equation 12 of that paper.

2.2 Environmental condition handling

In our framework, NEMO-PISCES handles environmental conditions. But availability of a nutrient in the environment does not mean that everything will be consumed, we are not modeling human, but single cell organisms. As we are working with fluxes a classical way to model such a mechanism is to add a pool. NEMO-PISCES will feed this pool with the availability of nutrient, the organism will be allowed to use as much nutrient as present, the excess of nutrient will be evacuate. As in the metabolic model, the environment pool respects the quasi-steady state approximations. For each metabolite in the pool we have a linear equation linking NEMO-PISCES input (blue arrow), exchange reactions with the metabolite network (black arrow) and the overflow of nutrient (red arrow). For instance, in the nitrate case, if we denote $v_p > 0$ (resp. $l_b < v_e < u_b$ and $v_o > 0$) as the input (resp. exchange and ovDOMerflow) flux of nitrate of NEMO-PISCES, we have the equation $0 = v_p + v_e - v_o$. l_b and u_b come from the thermodynamic constraints on the exchange reaction. A positive flux v_p brings nitrate in the environment pool, whereas a positive flux v_o remove nitrate

Table S2: Model parameters for phytoplankton with their default values in PISCES. When one value only is specified, this applies to all phytoplankton groups. Otherwise, the values correspond to picophytoplankton, nanophytoplankton and diatoms, respectively.

Parameter	Units	Value	Description
V_C^{max}	d^{-1}	0.83, 1.1, 1.1	Max gross growth rate at 0°C
V_N^{max}	$mol\,N\,(mol\,C)^{-1}\,d^{-1}$	0.145, 0.31, 0.35	Max N uptake rate at 0°C
V_P^{max}	$mol\,P\,(mol\,C)^{-1}d^{-1}$	0.0145, 0.031, 0.035	Max P uptake rate at 0°C
V_{Fe}^{max}	$\mu mol\,Fe\,(mol\,C)^{-1}d^{-1}$	88, 194, 220	Max Fe uptake rate at 0°C
α^i	$(W/m^2)^{-1}d^{-1}g\,C\,(g\,Chl)^{-1}$	4	initial slope of P/-I curve
b	–	1.066	Temperature sensitivity of growth
ξ	$g\,C\,(g\,Chl)^{-1}$	7.7	C associated with Chl
K_{NO3}	$\mu mol\,N\,L^{-1}$	0.1, 0.4, 0.65	Potential half-saturation constant for NO3
K_{NH4}	$\mu mol\,N\,L^{-1}$	0.03, 0.13, 0.22	Potential half-saturation constant for NH4
K_{PO4}	$\mu mol\,P\,L^{-1}$	0.006, 0.024, 0.04	Potential half-saturation constant for PO4
K_{Fe}	$nmol\,Fe\,L^{-1}$	1, 3, 4.5	Potential half-saturation constant for Fe
K_{Si}	$\mu mol\,Si\,L^{-1}$	8	Minimum half-saturation constant for silicate
K_S	$\mu mol\,Si\,L^{-1}$	20	Parameter for the half-saturation constant

Table S3: Model parameters for phytoplankton with their default values in PISCES. When one value only is specified, this applies to all phytoplankton groups. Otherwise, the values correspond to picophytoplankton, nanophytoplankton and diatoms, respectively.

Parameter	Units	Value	Description
$(\frac{C}{N})_0$	mol P mol N^{-1}	7.625	standard C/N Redfield ratio
Q_N^{min}	mol N mol C^{-1}	0.13, 0.08, 0.07	Minimum N/C quota of phytoplankton
Q_N^{max}	mol N mol C^{-1}	0.18, 0.16, 0.16	Maximum N/C quota of phytoplankton
$Q_{P,min}^i$	mmol P mol C^{-1}	1.6, 2, 2	Minimum P/C quota of phytoplankton
$Q_{P,max}^i$	mmol P mol C^{-1}	9.8, 11, 13	Maximum P/C quota of phytoplankton
$Q_{Fe,opt}^i$	µmol Fe mol C^{-1}	7	Optimal iron quota
$Q_{Fe,max}^i$	µmol Fe mol C^{-1}	80	Maximum iron quota

from the pool. Metabolic model conventions dictate that a negative flux of v_e bring the metabolite in the model, removing it from the pool. In our framework v_p is fixed, by NEMO-PISCES. But as we model an environment pool with an overflow reaction, we allow the exchange reaction to vary such that if a higher growth rate can be obtain with a smaller uptake of nitrate (i.e. not equal to the input of NEMO-PISCES) it can be achieve *i.e.* $-v_p \leq v_e \leq u_b$.

2.3 Use of GSMs with simulated environmental conditions

Input fluxes in NEMO-PISCES are in $molA.molC^{-1}.s^{-1}$ where A stands for a given metabolite. In a metabolic network, all fluxes are in $mmolA.gDW^{-1}.h^{-1}$ where gDW is a gram of dry weight of the considered organism, and A is a metabolite involved in the considered reaction. Thus, we must convert NEMO-PISCES flux for further use in a GSM as formulated above. Let us denote f_p the unit flux in NEMO-PISCES units and f_g the unit flux in GSM units, we are going to determine k such that $f_g = k.f_p$. We need two parameters: the molar mass of the organism's biomass (M_o in $kg.mol^{-1}$) and its composition in carbon (C_c in $molC.mol^{-1}$). Then $1kgDW$ of the organism is equivalent to $\frac{C_c}{M_o}molC$. When converting mol in $mmol$ and kg in g, the two factors cancel each other. It is necessary to transform seconds into hours with a factor of 3600. Thus we have :

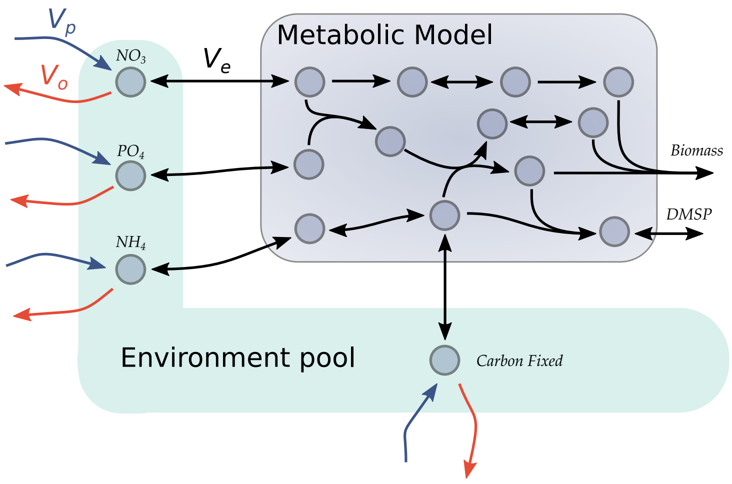

Figure S3: Schematic representation of the fluxes. Blue arrows represent fluxes fixed by the ESM. Black arrows represent the nutrients used by the GSM. Red arrows represent the nutrients not used by the GSM that need to be removed from the environment pool for the sake of steady state. In the future these red arrows should be connected to the ESM to represent feedback of the GSM on the environment.

$$f_g = 3600.\frac{C_c}{M_o}.f_p \quad .$$

The growth rate in NEMO-PISCES is in $molC.molC^{-1}.s^{-1}$, whereas in $mmolBiomass.gDW^{-1}.h^{-1}$ for GSM. Thus, with a similar procedure, we can convert one growth rate to another via:

$$growth_{GSM} = 3600.M_o.growth_{PISCES} \quad .$$

In most GSMs the molar mass of the biomass is normalized and we have $M_o = 1kg.mol^{-1} = 1g.mmol^{-1}$

2.4 Modeling perspectives for a fully coupled genome-enhanced ESM

Numerous scientific challenges persist in developing a fully integrated genome-enhanced ESM. Initially, it is essential to achieve more effective integration of growth metrics derived from GSMs within ESM tracers, along with translating the GSM's growth rates as organismal abundance. The subsequent phase involves better propagating uptake metrics employed by GSM within ESM tracers. Indeed, while ESM's fluxes indicate bioavailability, they do not necessarily represent the

actual quantities of materials utilized as substrates by GSMs. Upon the precise quantification of the characteristics above, it will be feasible to incorporate biological features further.

It entails the introduction of ESM's additional tracers governed by the dynamics established by GSMs. These should consider the broader range of nutrients produced or consumed by GSMs, as currently, only four nutrients (light, NO_3, NH_4, HPO_4) are accounted for. For example, one might consider dissolved organic carbon (DOC) metabolites (*18*) or other metabolites essential for trophic interactions or micronutrients (*47*). Expanding the modeling of organism distributions through existing GSMs is also crucial for ecological sake. Several GSMs are presently accessible, such as numerous heterotrophic bacteria, diatoms, and nano-phytoplankton, but this modeling endeavor could further inspire the development of new GSMs for capturing different ecotypes of a given species (*56*) or other emblematic planktonic organisms like *Calanoid*.

Appendix 3: Computational Pipeline

Data used for this study can be found on zenodo (DOI: 10.5281/zenodo.13621818)
https://zenodo.org/records/13621818
A Github repository with the developed code is available: https://github.com/Buiboni/MiNiMo

Data used in the study are:

- Environmental data are coming from PISCES.
- Genome-scale models of *Prochloroccocus MED4*, *Synechococcus sp. PCC 7002*, *Prochloroccocus* pangenome 'super-organism', *Thalassiosira pseudonana* and *Phaeodactylum tricornutum*.
- Niche description of the previous organisms.

3.1 Genome-scale models

Original metabolic genome-scale models can be found at:

- *Prochloroccocus MED4* (*iSO595*):
  https://github.com/segrelab/Prochlorococcus_Model.
- *Synechococcus sp. PCC 7002* (*iSyp728*):
  supplementary data of Hendry et al. 2016 (*23*).
- *Prochloroccocus* pangenome:
  https://github.com/jrcasey/PanGEM.
- *Thalassiosira pseudonana* (*iTps1432*): as available in the supplementary material from Tol, H. M. van & Armbrust, E. V. G (2021).
- *Phaeodactylum tricornutum* (*iLB1034*): as available in the supplementary material from Broddrick, J. T. et al. (2019).

3.2 Niche description

The niche description was computed through the script provided by (*21*) and available at: `https://gitlab.univ-nantes.fr/aregimbeau/metabolic-niche`.

From the output file, we need to run lrs, to have the H-representation of the niche. The equations are then used in a python script to produce estimations.

Appendix 4: Validation of the Genome-enabled ESM

4.1 Alignment and divergences between GSM and ESM predictions

Overall, GSM replicates quantitatively growth rate estimates from ESM (see Fig. 1 and below Supplementary Materials 7 for diatoms), and this accuracy increases when considering more ecotypes than only one strain (see below Supplementary Materials 5.4). However, for the sake of transparency, we also identified a few divergences that are worth mentioning (see fig. S4). These divergences mainly occur in front of provinces delimited by drastic variations of nutrients. These delimitations mostly follow boundary of areas of lipid and glycogen productions. This misalignment in physical or physiological fronts between modelings is encouraging as it pinpoints the need for better parameterizing the ESM at mesoscale or, complementary, building dedicated GSM suitable to follow fine *in situ* gradients of nutrients, as promoted by recent initiatives (https://biogeoscapes.org).

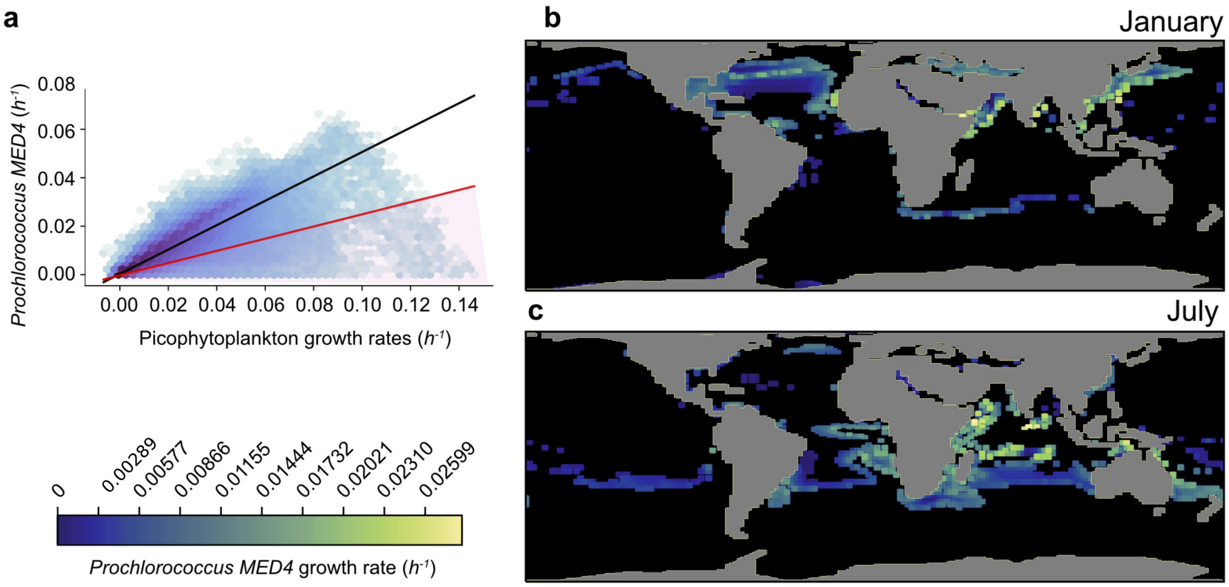

Figure S4: Identification of divergences between *Prochloroccocus MED4* and PISCES generic picophytoplankton growth rates. a |Comparison between *Prochlorococcus* MED4 and NEMO-PISCES generic picophytoplankton growth rate across times and depths. Red area delimits estimations far from the regression. As the regression follows the equation $y = slope * x + inter$ the area is identified by $y < slope/2 * x - inter$ with $slope = 0.45$ and $inter = 0.0003$. The red area covers less than 1% of the total estimation and 7% of the surface estimations. **b** |Localization of divergent surface growth rate estimation (5m depth) in January. **c** |Localization of divergent surface growth rate estimation (5m depth) in July.

The strong linear correlation between GSM and PISCES predictions results from multiple factors: the constraint on the uptake rates and the fact that we are computing growth rates offline. The offline computation "reinitialized" the error we could make at each time step (e.g., we use fluxes solely computed by NEMO-PISCES). The natural follow-up of this work is to implement explicit GSM feedback in NEMO-PISCES. This would then allow us to compare abundances, yielding less linearly linked results. At each time step, the fluxes will be influenced by the abundance, which will modify the growth and then the abundance of the next step. Only numerical simulation will tell us if the divergence shown here will worsen or will self-regulate.

4.2 Comparison with *in situ* data

ESMs are highly parameterized for generic classes of organisms (i.e., diatoms, picophytoplankton, and nanophytoplankton), and considering that we are modeling specific strains, the comparison between our prediction and *in situ* data remains quantitatively difficult. However, for validation sake, our predictions were compared with the AMT13 dataset obtained from Supplementary Material of Casey et al. (2022) (*13*). Quantitatively speaking, abiotic components, except for the ammonium concentration, are reasonably coherent (see fig. S5). NEMO-PISCES data represent a mean over the month of the sampling date of the AMT13 cruise.

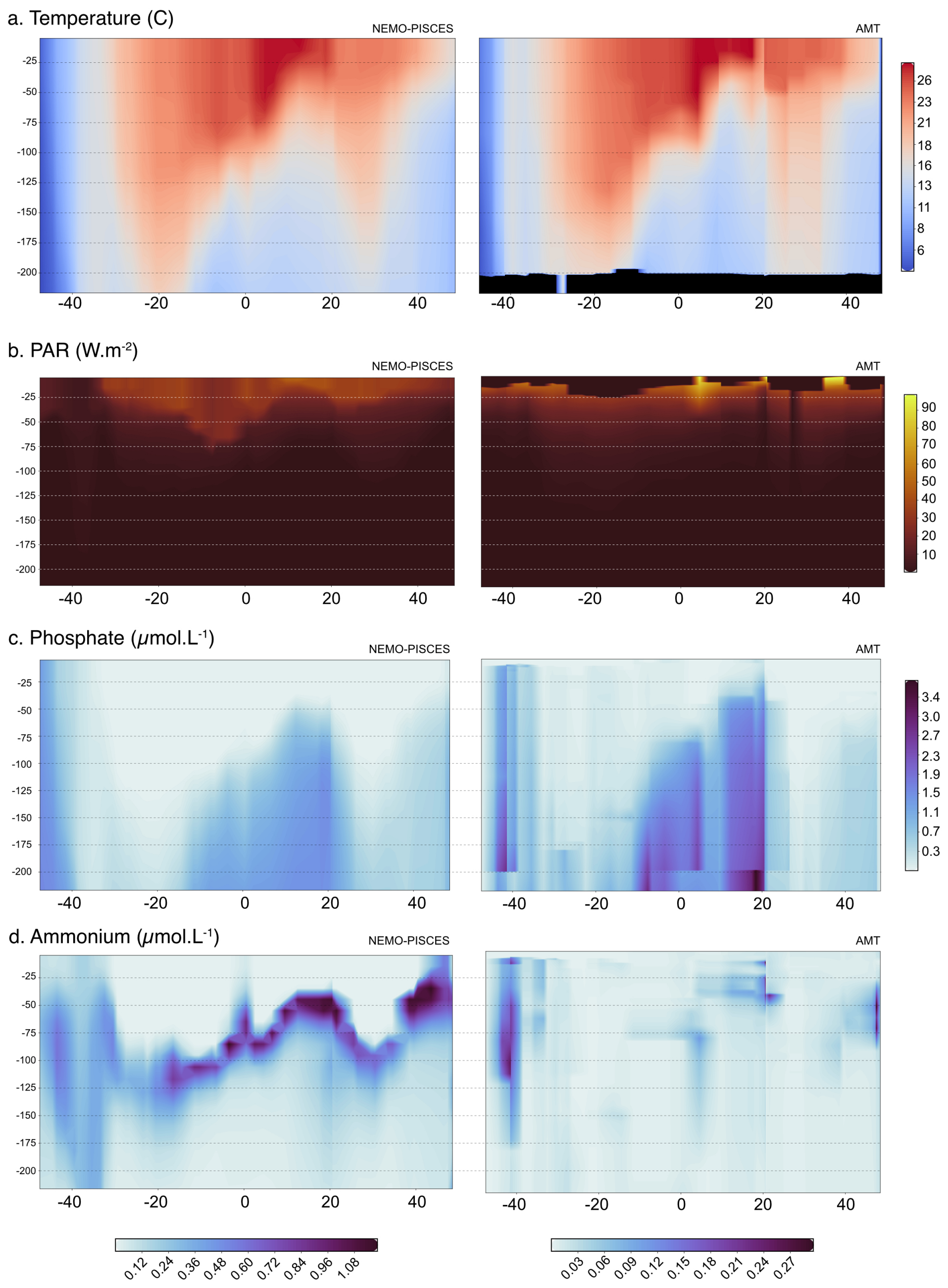

Figure S5: Comparison between NEMO-PISCES estimation of abiotic components and corresponding AMT13 *in situ* data. a |Temperature. **b** |PAR, the black area on the surface is due to the lack of data from the AMT13 cruise. **c** |Orthophosphate concentration. **d** |Ammonium concentration. The difference in magnitude was too high to represent both transects with the same scale.

Furthermore, as the *in situ* biological observations are not the same currencies as those used

for NEMO-PISCES estimations (AMT13 data are in cell count, NEMO-PISCES is in carbon concentration), , only these qualitative conclusions are accurate. We compared our growth estimation based on *Prochloroccocus MED4* GSM and its abundance in cell number (see fig. S6a), as well as the growth of picophytoplankton estimated by NEMO-PISCES in comparison to the abundance of picophytoplankton cells (see fig. S6b). We find coherent patterns, particularly the ”U-shape” in both comparisons, qualitatively validating our estimations with *in situ* data.

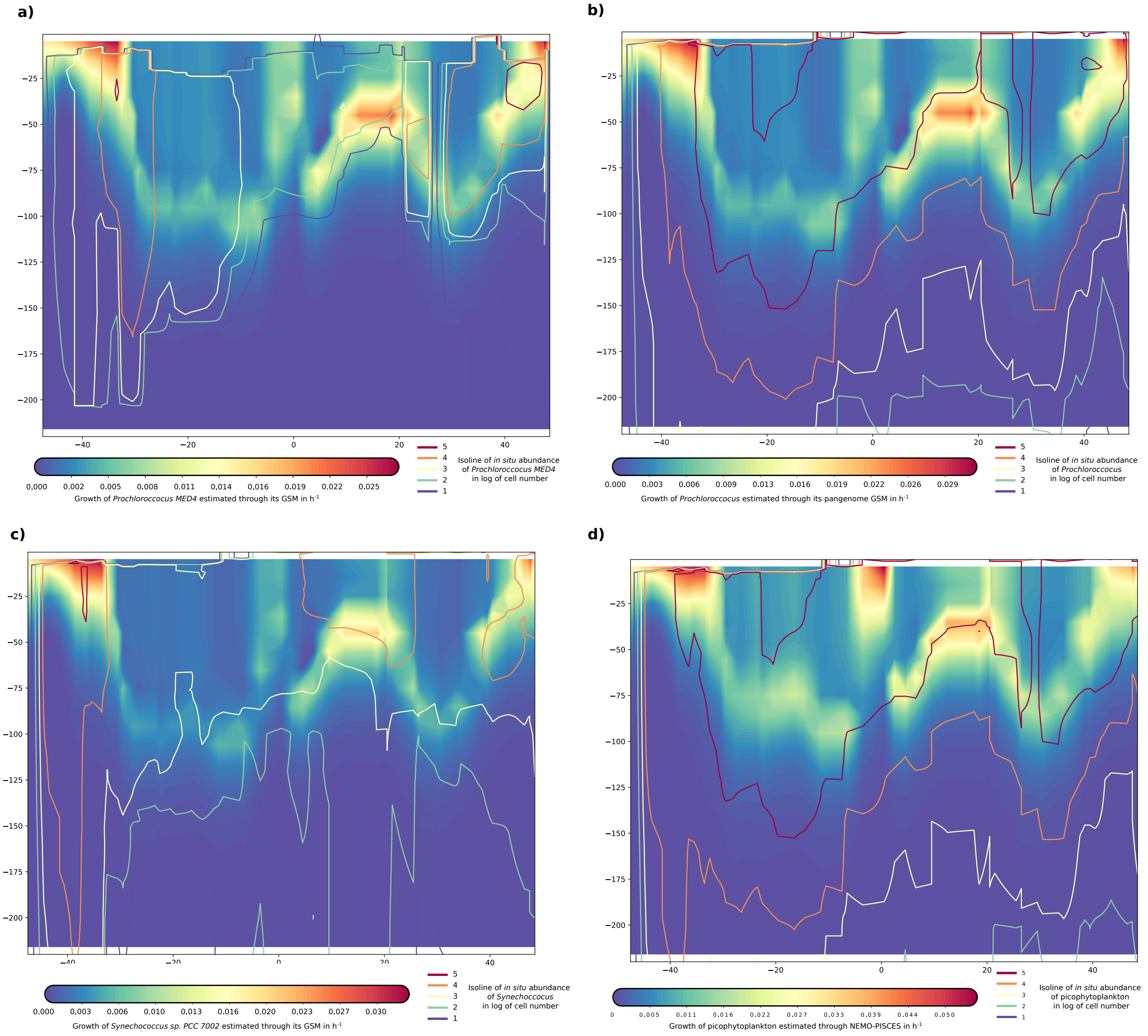

Figure S6: Comparison with AMT13 *in situ* data a |Comparison between *Prochloroccocus MED4* growth produced by our framework and the cell abundance of *Prochloroccocus MED4* represented by isocline of log values. **b** |Comparison between *Prochloroccocus* growth produced by our framework through the pangenome of *Prochloroccocus* and the cell abundance of *Prochloroccocus* represented by isocline of log values. **c** |Comparison between *Synechococcus sp. PCC 7002* growth produced by our framework and the cell abundance of *Synechoccocus* represented by isocline of log values. **d** |Comparison between picophytoplankton growth produced by NEMO-PISCES and the cell abundance represented by isoline of log values.

4.3 Comparison of resource constraints with available stress definitions

Our definition of resource constraints is driven by a mechanistic understanding of metabolic needs to sustain a given growth. Thus, this definition differs from stress definitions used in seminal studies by Saito et al. (2014) (*30*) (based on proteomic analysis) and Ustick et al. (2021) (*29*) (based on gene expression). Each definition of stress refers to distinct aspects of biological system plasticity and utilizes different data types. The stress proposed by Ustick et al. relies on semi-quantitative measurements and statistical modeling. In contrast, the stress described by Saito et al. is quantitative (proteins concentration) but does not consider rates or fluxes necessary for incorporating this knowledge into ESMs. It is worth noting that Saito et al.'s definition of stress is more closely related to resources metabolic constraints, but comparing these different definitions would require extensive additional work, including investigating the inner concentrations of nitrogen or iron in *Prochlorococcus MED4* (i.e., data not available to our knowledge at the time of this study).

Therefore, while aligning all proposed definitions of stress is of great interest, it is challenging and beyond the scope of our manuscript. Worth noticing, this topic remains an open question in Systems Biology, necessitating further clarification of the concepts of robustness and plasticity across biological data scales (*57–59*).

Appendix 5: Genome-enabled ESM limits

Genome-enabled ESM allows accurate predictions of several features but, as for all modelings, shows limitations that should guide future experiments.

5.1 GSM limitations

The GSM framework relies on the sole biochemical description of the biological system and solving its linear formulation. Thus, genome-scale modeling remains limited in reproducing biological specificities. For instance, GSM does not include gene or other post-transcriptional regulation, producing nonlinear responses. Therefore, GSM does not hold photoprotection mechanisms *per se*. As shown in fig. S8, estimations wrongly predict higher pigment productions in the surface ocean, where light is abundant, misaligning with in situ observations where photoprotection occurs. This invalidation calls for complementary modeling of this phenomenon, such as gene regulation, to better integrate the adaptative response of the organism to light. The need to incorporate nonlinear responses through gene regulation also holds for modeling the impact of temperature.

In its current form, the temperature does not affect the inner reaction of the model. Herein, the temperature influences only the uptake of nutrients and is handled by NEMO-PISCES. Integrating such nonlinear regulations requires further modeling works that are out of the scope of this manuscript. However, our current GSM modeling still indicates areas for improvements that could help design GSMs specific to ocean studies (e.g., see below complementary Supplementary Materials 6.1 about the current limitation to predict *Prochlorococcus MED4* pigments). Indeed, most of the available GSM models are developed following extensive *in vivo* experiments. It results in a GSM's biomass composition that reflects combinatorics of experimental stresses. In particular, recent studies (*13*) advocate for refining the biomass composition for better modeling *in situ* behavior or ecotypes (see Appendix 6.3).

5.2 ESM limitations

Genome-enabled ESM is limited by the diversity of nutrients described through the ESM, which could substantialy impact our result if we consider chemical elements that mediated interactions (*60*). In particular, while we introduced more functional diversity and molecular description through

GSMs, we remain dependent on only four nutrients (including light). Moreover, to model more diversity, we would need new parameters for light absorption (i.e., especially for modeling HL and LL *Prochloroccocus* clades).

It is worth noticing as we are only making offline computations, no effect of our computation is made on the original version of NEMO-PISCES.

5.3 Connection between GSM and ESM

Light is modeled through the quantity of carbon fixed. However, the model is free to use this carbon (as done with the other nutrients, see Material and Methods). In reality, organisms must adapt their cellular content to control the quantity of light they absorb and then control the amount of carbon they are fixing. Under excessive light exposure, the organisms must evacuate the energy through photoprotection. Long-term photoprotection can be achieved by adapting cellular composition, whereas the main short-term adaptation is through non-photochemical quenching, producing heat, and altering the organism's integrity. These mechanisms are not represented in our model.

Moreover, we need to emphasize the need of a common currency between the two types of model. We were able to adapt the flux of ESM (in $molX.molC^-1.s^-1$, where X is the considered nutrient) with the flux of the GSMs (in $mmolX.gDW^-1.h^-1$).

5.4 Better representation of picophytoplankton

GSMs are only representing one strain of the corresponding NEMO-PISCES group. By adding other ecotypes or approximation of Prochlorococcus communities, GSM predictions are more in line with ESM estimates. Thus, as shown in fig. S7, the cumulative sum of squared error decreases with the number of organisms we represent through GSMs. As the NEMO-PISCES model computes growth and fluxes per carbon unit when representing several organisms, we can expect the community to grow as much as the highest calculated growth. However, this crude community representation has its limits, mainly when representing a community that contains organisms with very different niches. For instance, let us consider a community of organisms A and B. The water mass will spend a certain amount of time in the niche of A, and the community will be composed of 90% of A. When the condition changes in favor of B, the highest growth computed will consider only a tiny

fraction of the community (10% in our example). Here, we can see that a crude representation of the community leads to an ever-growing community, in terms of carbon, while it can be stable in carbon, but can change in terms of organisms composition.

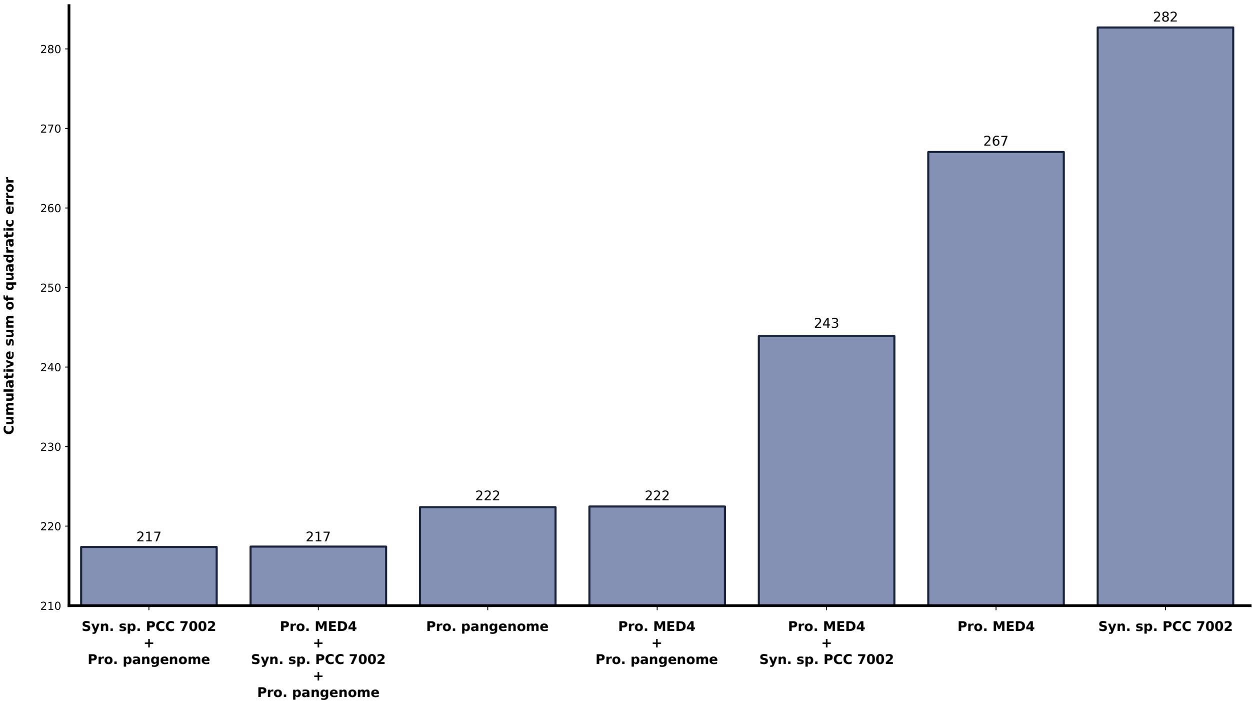

Figure S7: Impact of community modeling Cumulative sum of the quadratic error between GSMs growth and NEMO-PISCES picophytoplankton growth. When two or more GSMs are used, the growth rate used for comparison is the highest among those computed with the GSMs, estimations on 4 743 060 grid points (i.e., points where the growth outputted by NEMO-PISCES is over 0 and the temperature over $10°C$)

Appendix 6: *Prochloroccocus* MED4 supplementary discussions

6.1 Using *Prochlorococcus MED4*'s GSM to design ESM traits

Whereas standard ocean biogeochemical models propose different parameter values for a given organism to depict particular phenotypic traits (*8*) (i.e., parameterized contributions of macromolecules such as pigments, proteins, or lipids for biomass), the GSM approach characterizes the entire phenotypic plasticity of the organism per se, including its intracellular content. As an illustration, ESMs represent chlorophyll to carbon ratio through differential equation mainly based on light and nutrient limitation (*61*).

Our *Prochlorococcus MED4* GSM simulations show variations in pigment production across the global ocean (fig. S8a) and at different depths (fig. S8b) that differ markedly from growth rates (Fig. 1d). These estimations arise without specific parameterization and are consistent with results from Argo floats made at the community scale (*62*), showing a disconnect between abundance and pigment concentration. However, in the case of pigment production, we can see that our model is inconsistent with previous results. Indeed, the GSM does not model photoprotective processes, so the production of pigment is solely based on metabolic availability. Further work needs to be done to correctly represent the dissipative mechanism (*22*) in the next generation of GSM.

Similarly, we can use the GSM to explore how MED4 growth rates relate to the elements that constitute its biomass (fig. S8c). To do so, we compute the auxiliary flux that goes through different reactions responsible for macromolecule production. Then, we calculate the correlation between these flux values and their corresponding growth rate across all environmental conditions. Except for lipids and glycogen, we find a lack of correlations between growth rates and the ability of the organism to produce proteins, amino acids, or nucleic acids. The relative independence between growth rates and biomass constituents in *Prochlorococcus* is explained by acclimation strategies that would increase organismal fitness (*63*).

The molecular composition from GSMs can help develop improved ESMs as it indicates a new manner to link growth rate, metabolism, and cellular composition even in sub-optimal growth conditions. The importance of estimating the molecular composition has already been made in Casey et al. (*13*), showing the difference in molecular composition across cyanobacterial strains and its variation depending on environmental conditions. Our results explore macromolecular

concentrations absent from ESMs and help estimate molecular contents based on the metabolic abilities of the organisms that are classically used in ESMs, rather than on extensive integration of experimental data.

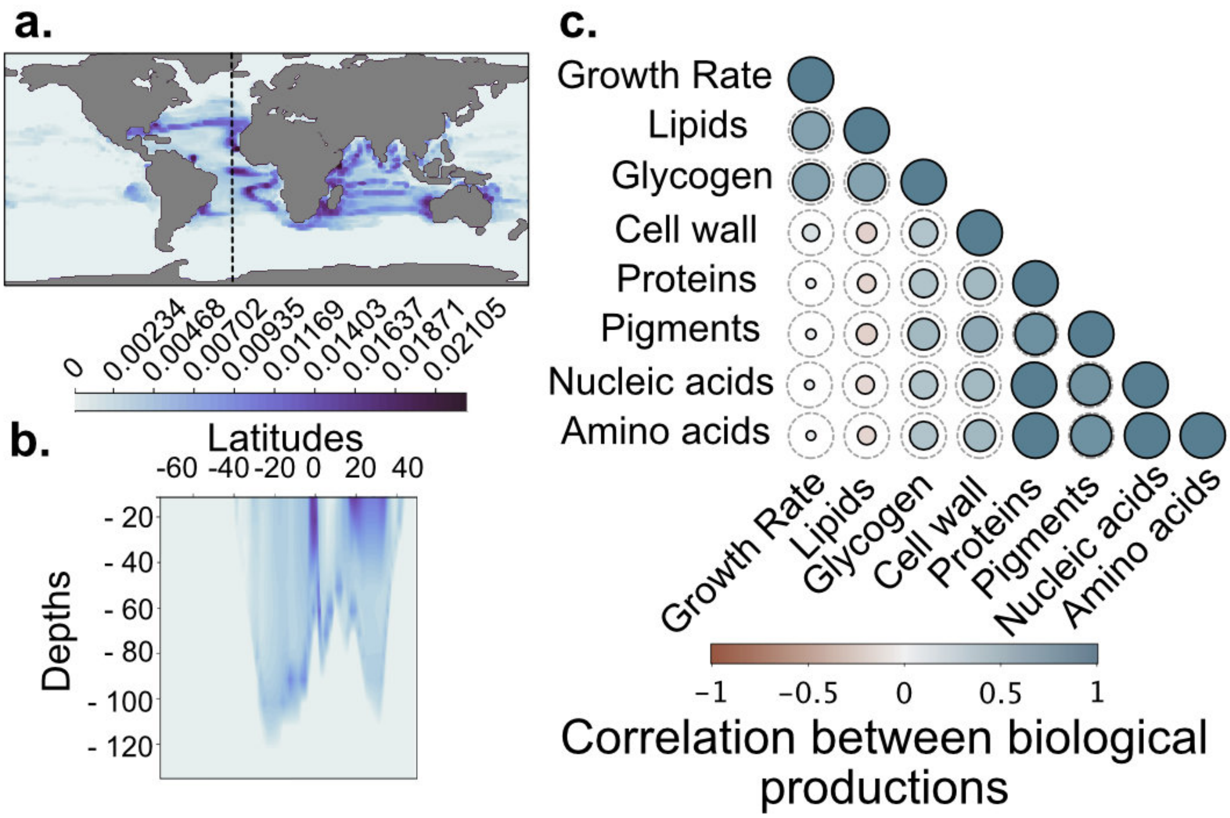

Figure S8: Estimating *Prochlorococcus MED4* pigment production and other components of the cellular composition to be used in ESM. **a** |Description of global ocean pigment production at 5 m depth in $mol_{pigment}.mol^{-1}_{biomass}.h^{-1}$. The dashed black line describes the transect highlighted in the lower panel. **b** |Distribution of *Prochlorococcus MED4* pigment across depths and latitudes at longitude -24°. **c** |Correlogram of estimated *Prochlorococcus MED4* growth rates and cellular composition across the global ocean (more than 10^6 estimations). Pairwise correlation scores investigate the relationship between growth rates and biological components' overproductions constitutive of the biomass composition. For reference, dashed circles describe absolute correlations equal to one, and colors show the correlation value as bold circle widths proportional to the corresponding pairwise correlation. No circle indicates a correlation close to zero.

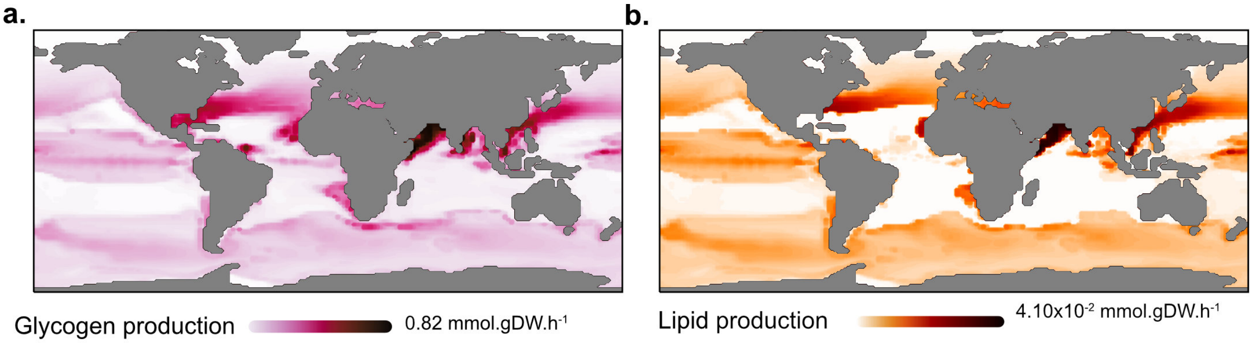

Figure S9: Estimating *Prochlorococcus MED4* lipid and glycogen production. **a** |Glycogen production by *Prochloroccocus MED4* across the surface ocean in January. **b** |Lipid production by *Prochloroccocus MED4* across the surface ocean in January.

6.2 Estimates of lipids in *Prochloroccocus* MED4

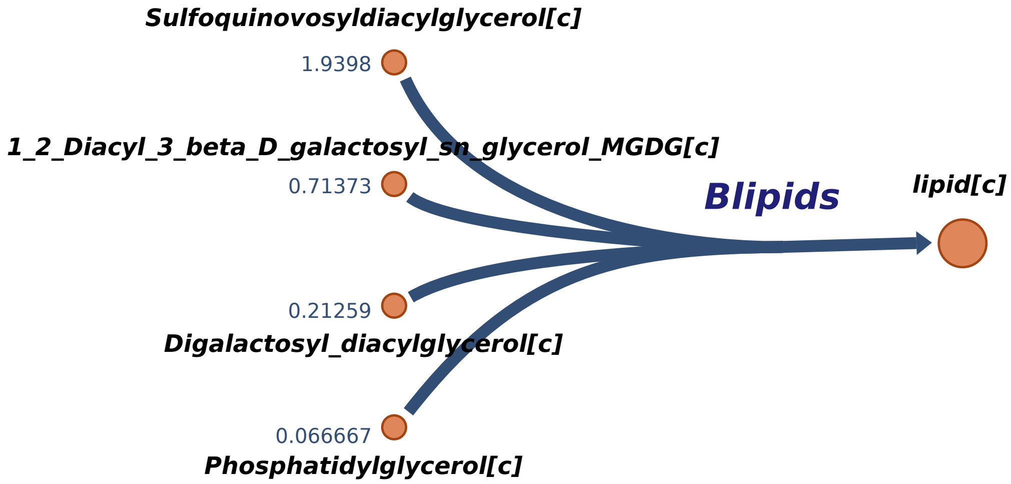

Figure S10: Metabolic reaction responsible for the production of lipids in the model iSO595 (produced with Escher)

The lipids considered here are a combination of different lipid metabolites, including one phospholipid (i.e., Phosphatidylglycerol, 1, 2-diacyl-3-β-D-galactosyl-sn-glycerol, Digalactosyldiacylglycerol, and Sulfoquinovosyldiacylglycerol). This composition can explain the difference in carbon storage when *Prochloroccocus* MED4 growth is limited by phosphorus (fig. S11).

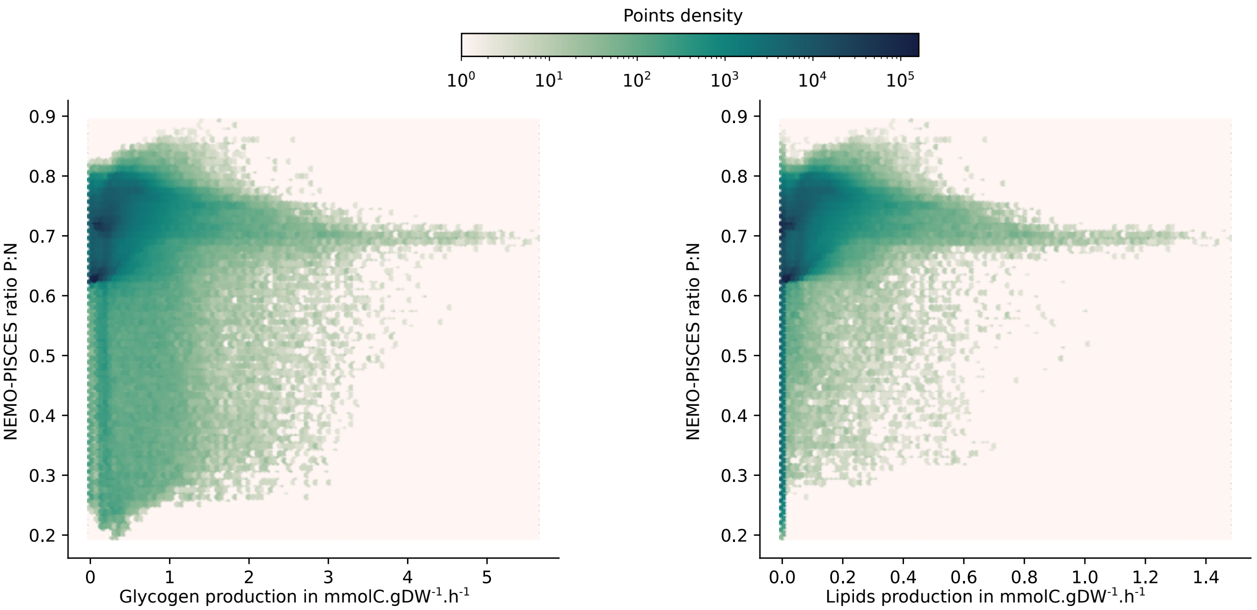

Figure S11: Carbon storage depending on P:N ratio The Left panel shows the quantity of carbon stored through glycogen, and the right panel shows the carbon stored through lipids. It worth noting that the lipids represented here are the over production of lipids, that is we did not take into account the amount of lipids included in the biomass reaction. Glycogen, on the other hand, is not included in the biomass reaction.

We postulate that the difference in term of quantity of carbon fixed through either of the two compounds is due to ATP uses. We quantified the amount of carbon fixed through photosynthesis necessary to produce an equivalent amount of carbon in the form of lipids or glycogen. Our findings indicate that storing carbon in the form of lipids requires seven times more carbon fixation than storing it as glycogen.

6.3 Iron in *Prochloroccocus* MED4

Iron is present in *Prochloroccocus* MED4 GSM. However, when used in NEMO-PISCES, it results in a very small growth rate, half that without considering the iron flux. A regression between NEMO-PISCES picophytoplankton growth rate and *Prochloroccocus* MED4 GSM without iron yields a slope of 0.45 and an *r-value* of 0.88, while doing the same regression with the GSM considering iron gives us a slope of 0.23 and an $rvalue$ of 0.75.

Using the GSM with iron only explains a quarter of the NEMO-PISCES picophytoplankton

growth rate. One reason for this is the very high iron quota in the GSM compared to NEMO-PISCES. NEMO-PISCES allows a variable iron quota with a maximum (iron to carbon ratio) of 40 μmolFe.molC^{-1}. However, the fixed ratio of iron to carbon of the GSM biomass is over 86 μmolFe.molC^{-1}, more than twice the maximum ratio allowed in NEMO-PISCES. Such a difference in how iron is integrated into the models could be why we observe such variable results. This observation is another motivation for designing specific GSMs for biogeochemical modeling purposes. Such a high fixed value of iron quota for the biomass composition does not represent the organism in its natural environment.

Appendix 7: Diatoms in the global ocean

For reproducibility, we performed similar GSM modelings on two diatoms (*Phaeodactylum tricornutum* (*25*) and *Thalassiosira pseudonana* (*24*)), for which metabolic models are available. As with *Prochlorococcus MED4* growth rate simulations, we consider the same nutrients plus nitrate and silicate (only for *T. pseudonana*).

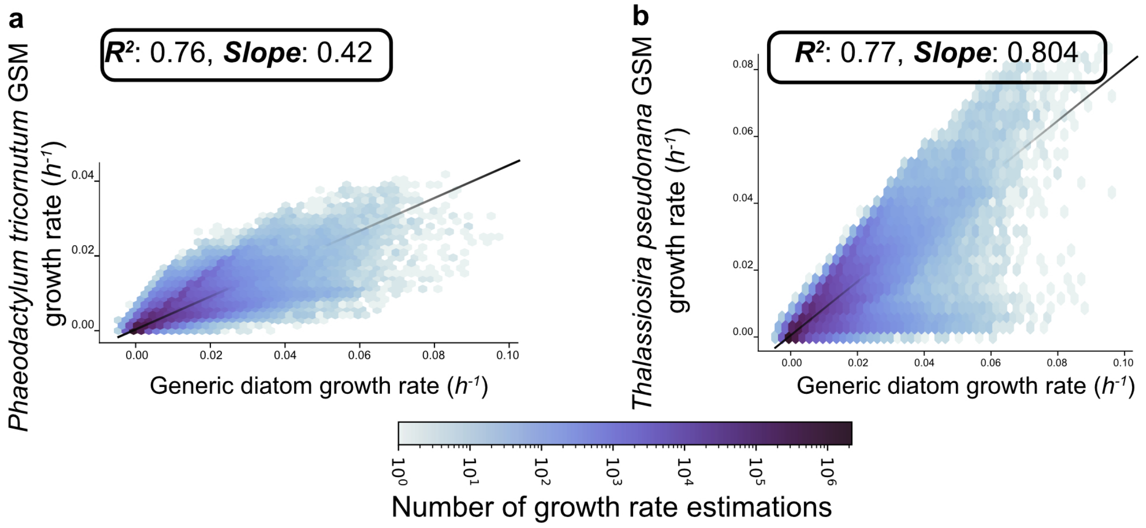

Figure S12: Comparison between growth rates estimated from PISCES generic diatom and two genome-scale models. a |Relationship between growth rates of *Phaeodactylum tricornutum* and PISCES generic diatom across space and time. **b** |Relationship between growth rates of *Thalassiosira pseudonana* and PISCES generic diatom across space and time.

Both GSMs show different metabolic networks (resp. 2162 and 6079 reactions for *P. tricornutum*, and *T. pseudonana*), expressing different metabolic needs. Both qualitatively replicate the growth rates of a generic diatom (see fig. S12, fig. S13a). Across space and time, GSM simulations are correlated with PISCES estimation ($r > 0.86$), and the slope is higher for *T. pseudonana* (i.e., 0.8) than for *P. tricornutum* (i.e., 0.42)(see fig. S12). However, the GSM framework allows a comparison of the individual growth rate of both diatom strains across space and time. It confirms the extensive range of *T. pseudonana* growth rate magnitude and its ubiquity compared to *P. tricornutum* (fig. S13b). The linear regression between the two growth rates exhibits an almost two-fold higher growth rate for *T. pseudonana*. This result is supported by the fact that *P. tricornutum* is not as abundant in the ocean as *T. pseudonana* (*12, 64*).

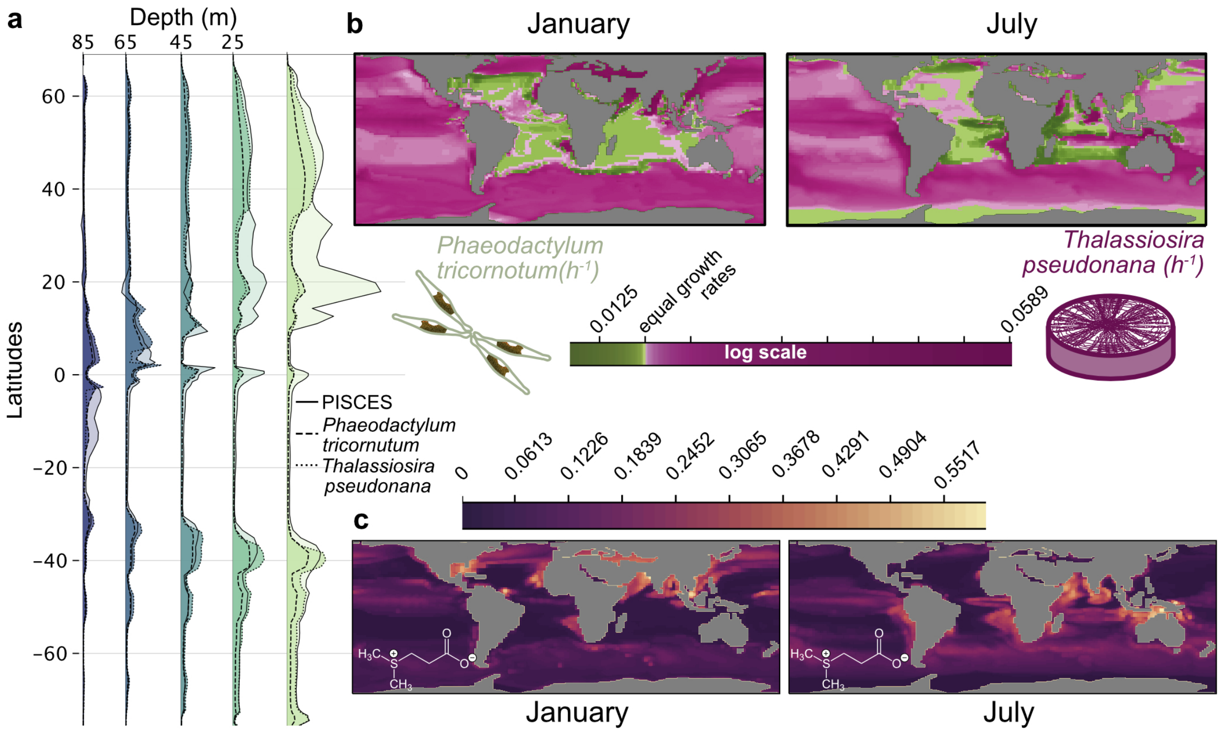

Figure S13: Comparison between *Phaeodactylum tricornutum* and *Thalassiosira pseudonana* growth rates and *Phaeodactylum tricornutum* implication in the DMSP production. a |Growth rates (h^{-1}) distribution of PISCES generic diatom and two GSMs across latitudes and depths at longitude 24. **b** |Relative growth rates of both GSMs in January (left panel) and July (right panel) across the surface ocean. **c** |Production of dimethylsulfoniopropionate (DMSP) by *Phaeodactylum tricornutum* in January (left panel) and July (right panel) across the surface ocean. DMSP production rate is in $mol_{DMSP}.mol_{biomass}^{-1}.h^{-1}$

We can also estimate flux for metabolites challenging to measure, such as DiMethyl SulfonioPropionate - DMSP (fig. S13c and fig. S14b). In particular, we show a seasonality of diatoms DMSP's production on the ocean's surface and its increase with light exposure in the study of *T. pseudonana* (*65*) (correlation of $r < -0.9$ with light constraint fig. S14). Similar estimation would have required extensive parameterizing efforts in ESMs for predicting a metabolite of importance in microbial ecology (*66*) and climatic prediction (*67*).

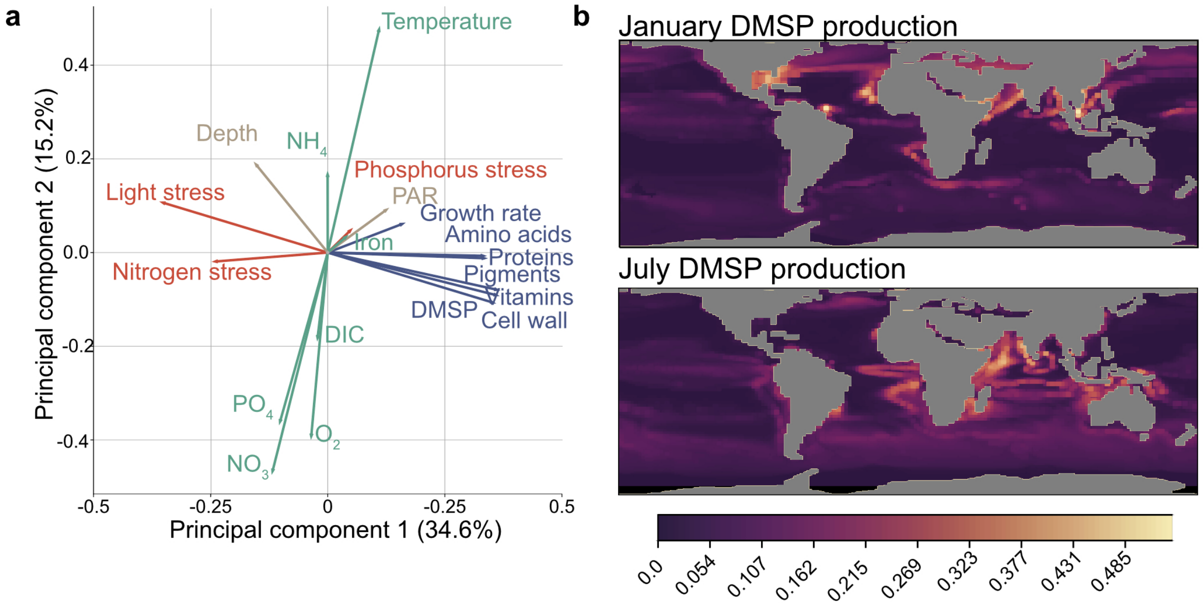

Figure S14: Simulation of *Thalassiosira pseudonana* genome-scale model across the global ocean. a |Principal component analysis on more than 10^7 samples across the global ocean over a year. Physiological factors emphasized by metabolic modeling are in blue for organismal composition and red for constraints. Environmental factors are in green and biogeography parameters are in grey. **b** |Production of dimethylsulfoniopropionate (DMSP) by *Thalassiosira pseudonana* in January (top panel) and July (bottom panel) across the surface ocean. DMSP production rate is in $mol_{DMSP}.mol_{biomass}{}^{-1}.h^{-1}$

REFERENCES AND NOTES

1. P. Friedlingstein, M. O'Sullivan, M. W. Jones, R. M. Andrew, J. Hauck, A. Olsen, G. P. Peters, W. Peters, J. Pongratz, S. Sitch, C. le Quéré, J. G. Canadell, P. Ciais, R. B. Jackson, S. Alin, L. E. O. C. Aragão, A. Arneth, V. Arora, N. R. Bates, M. Becker, A. Benoit-Cattin, H. C. Bittig, L. Bopp, S. Bultan, N. Chandra, F. Chevallier, L. P. Chini, W. Evans, L. Florentie, P. M. Forster, T. Gasser, M. Gehlen, D. Gilfillan, T. Gkritzalis, L. Gregor, N. Gruber, I. Harris, K. Hartung, V. Haverd, R. A. Houghton, T. Ilyina, A. K. Jain, E. Joetzjer, K. Kadono, E. Kato, V. Kitidis, J. I. Korsbakken, P. Landschützer, N. Lefèvre, A. Lenton, S. Lienert, Z. Liu, D. Lombardozzi, G. Marland, N. Metzl, D. R. Munro, J. E. M. S. Nabel, S. I. Nakaoka, Y. Niwa, K. O'Brien, T. Ono, P. I. Palmer, D. Pierrot, B. Poulter, L. Resplandy, E. Robertson, C. Rödenbeck, J. Schwinger, R. Séférian, I. Skjelvan, A. J. P. Smith, A. J. Sutton, T. Tanhua, P. P. Tans, H. Tian, B. Tilbrook, G. van der Werf, N. Vuichard, A. P. Walker, R. Wanninkhof, A. J. Watson, D. Willis, A. J. Wiltshire, W. Yuan, X. Yue, S. Zaehle, Global carbon budget 2020. *Earth Syst. Sci. Data* **12**, 3269–3340 (2020).

2. M. J. Follows, S. Dutkiewicz, Modeling diverse communities of marine microbes. *Ann. Rev. Mar. Sci.* **3**, 427–451 (2011).

3. K. Fennel, J. P. Mattern, S. C. Doney, L. Bopp, A. M. Moore, B. Wang, L. Yu Ocean biogeochemical modelling. *Nat. Rev. Methods Primers* **2**, 76 (2022).

4. O. Aumont, C. Ethé, A. Tagliabue, L. Bopp, M. Gehlen, PISCES-v2: An ocean biogeochemical model for carbon and ecosystem studies. *Geosci. Model Dev.* **8**, 2465–2513 (2015).

5. B. A. Ward, S. Dutkiewicz, O. Jahn, M. J. Follows, A size-structured food-web model for the global ocean. *Limnol. Oceanogr.* **57**, 1877–1891 (2012).

6. J. Monod, *Recherches sur la croissance des cultures bactériennes* (Hermann & Cie, 1942).

7. M. R. Droop, The nutrient status of algal cells in continuous culture. *J. Mar. Biol. Assoc. U.K.* **54**, 825–855 (1974).

8. C. Le Quéré, S. P. Harrison, I. Colin Prentice, E. T. Buitenhuis, O. Aumont, L. Bopp, H. Claustre, L. Cotrim da Cunha, R. Geider, X. Giraud, C. Klaas, K. E. Kohfeld, L. Legendre, M. Manizza, T. Platt, R. B.

Rivkin, S. Sathyendranath, J. Uitz, A. J. Watson, D. Wolf-Gladrow, Ecosystem dynamics based on plankton functional types for global ocean biogeochemistry models. *Glob. Chang. Biol.* **11**, 2016–2040 (2005).

9. A. W. Omta, J. D. Liefer, Z. V. Finkel, A. J. Irwin, D. Sher, M. J. Follows, A model of time-dependent macromolecular and elemental composition of phytoplankton. *J. Theor. Biol.* **592**, 111883 (2024).

10. V. J. Coles, M. R. Stukel, M. T. Brooks, A. Burd, B. C. Crump, M. A. Moran, J. H. Paul, B. M. Satinsky, P. L. Yager, B. L. Zielinski, R. R. Hood, Ocean biogeochemistry modeled with emergent trait-based genomics. *Science* **358**, 1149–1154 (2017).

11. D. D'Alelio, D. Eveillard, V. J. Coles, L. Caputi, M. R. d'Alcala, D. Iudicone, Modelling the complexity of plankton communities exploiting omics potential: From present challenges to an integrative pipeline. *Curr. Opin. Syst. Biol.* **13**, 68–74 (2019).

12. S. Sunagawa, S. G. Acinas, P. Bork, C. Bowler, Tara Oceans Coordinators, S. G. Acinas, M. Babin, P. Bork, E. Boss, C. Bowler, G. Cochrane, C. de Vargas, M. Follows, G. Gorsky, N. Grimsley, L. Guidi, P. Hingamp, D. Iudicone, O. Jaillon, S. Kandels, L. Karp-Boss, E. Karsenti, M. Lescot, F. Not, H. Ogata, S. Pesant, N. Poulton, J. Raes, C. Sardet, M. Sieracki, S. Speich, L. Stemmann, M. B. Sullivan, S. Sunagawa, P. Wincker, D. Eveillard, G. Gorsky, L. Guidi, D. Iudicone, E. Karsenti, F. Lombard, H. Ogata, S. Pesant, M. B. Sullivan, P. Wincker, C. de Vargas, *Tara* Oceans: Towards global ocean ecosystems biology. *Nat. Rev. Microbiol.* **18**, 428–445 (2020).

13. J. R. Casey, R. M. Boiteau, M. K. M. Engqvist, Z. V. Finkel, G. Li, J. Liefer, C. L. Müller, N. Muñoz, M. J. Follows, Basin-scale biogeography of marine phytoplankton reflects cellular-scale optimization of metabolism and physiology. *Sci. Adv.* **8**, eabl4930 (2022).

14. C. Gu, G. B. Kim, W. J. Kim, H. U. Kim, S. Y. Lee, Current status and applications of genome-scale metabolic models. *Genome Biol.* **20**, 121 (2019).

15. A. Bordbar, J. M. Monk, Z. A. King, B. O. Palsson, Constraint-based models predict metabolic and associated cellular functions. *Nat. Rev. Genet.* **15**, 107–120 (2014).

16. X. Fang, C. J. Lloyd, B. O. Palsson, Reconstructing organisms in silico: Genome-scale models and their emerging applications. *Nat. Rev. Microbiol.* **18**, 731–743 (2020).

17. A. R. Pacheco, M. Moel, D. Segrè, Costless metabolic secretions as drivers of interspecies interactions in microbial ecosystems. *Nat. Commun.* **10**, 103 (2019).

18. M. A. Moran, E. B. Kujawinski, W. F. Schroer, S. A. Amin, N. R. Bates, E. M. Bertrand, R. Braakman, C. T. Brown, M. W. Covert, S. C. Doney, S. T. Dyhrman, A. S. Edison, A. M. Eren, N. M. Levine, L. Li, A. C. Ross, M. A. Saito, A. E. Santoro, D. Segrè, A. Shade, M. B. Sullivan, A. Vardi Microbial metabolites in the marine carbon cycle. *Nat. Microbiol.* **7**, 508–523 (2022).

19. A. Tagliabue, 'Oceans are hugely complex': Modelling marine microbes is key to climate forecasts. *Nature* **623**, 250–252 (2023).

20. L. Kwiatkowski, O. Aumont, L. Bopp, P. Ciais, The impact of variable phytoplankton stoichiometry on projections of primary production, food quality, and carbon uptake in the global ocean. *Global Biogeochem. Cycles* **32**, 516–528 (2018).

21. A. Régimbeau, M. Budinich, A. Larhlimi, J. J. Pierella Karlusich, O. Aumont, L. Memery, C. Bowler, D. Eveillard, Contribution of genome-scale metabolic modelling to niche theory. *Ecol. Lett.* **25**, 1352–1364 (2022).

22. S. Ofaim, S. Sulheim, E. Almaas, D. Sher, D. Segrè, Dynamic allocation of carbon storage and nutrient-dependent exudation in a revised genome-scale model of prochlorococcus. *Front. Genet.* **12**, 91 (2021).

23. J. I. Hendry, C. B. Prasannan, A. Joshi, S. Dasgupta, P. P. Wangikar, Metabolic model of *Synechococcus* sp. PCC 7002: Prediction of flux distribution and network modification for enhanced biofuel production. *Bioresour. Technol.* **213**, 190–197 (2016).

24. H. M. van Tol, E. V. Armbrust, Genome-scale metabolic model of the diatom *Thalassiosira pseudonana* highlights the importance of nitrogen and sulfur metabolism in redox balance. *PLOS ONE* **16**, e0241960 (2021).

25. J. T. Broddrick, N. du, S. R. Smith, Y. Tsuji, D. Jallet, M. A. Ware, G. Peers, Y. Matsuda, C. L. Dupont, B. G. Mitchell, B. O. Palsson, A. E. Allen, Cross-compartment metabolic coupling enables flexible photoprotective mechanisms in the diatom *Phaeodactylum tricornutum*. *New Phytol.* **222**, 1364–1379 (2019).

26. Z. I. Johnson, E. R. Zinser, A. Coe, N. P. McNulty, E. M. S. Woodward, S. W. Chisholm, Niche partitioning among *Prochlorococcus* ecotypes along ocean-scale environmental gradients. *Science* **311**, 1737–1740 (2006).

27. J. A. Sohm, N. A. Ahlgren, Z. J. Thomson, C. Williams, J. W. Moffett, M. A. Saito, E. A. Webb, G. Rocap, Co-occurring *Synechococcus* ecotypes occupy four major oceanic regimes defined by temperature, macronutrients and iron. *ISME J.* **10**, 333–345 (2015).

28. A. C. Martiny, G. I. Hagstrom, T. De Vries, R. T. Letscher, G. L. Britten, C. A. Garcia, E. Galbraith, D. Karl, S. A. Levin, M. W. Lomas, A. R. Moreno, D. Talmy, W. Wang, K. Matsumoto, Marine phytoplankton resilience may moderate oligotrophic ecosystem responses and biogeochemical feedbacks to climate change. *Limnol. Oceanogr.* **67**, S378–S389 (2022).

29. L. J. Ustick, A. A. Larkin, C. A. Garcia, N. S. Garcia, M. L. Brock, J. A. Lee, N. A. Wiseman, J. K. Moore, A. C. Martiny, Metagenomic analysis reveals global-scale patterns of ocean nutrient limitation. *Science* **372**, 287–291 (2021).

30. M. A. Saito, M. R. McIlvin, D. M. Moran, T. J. Goepfert, G. R. DiTullio, A. F. Post, C. H. Lamborg, Multiple nutrient stresses at intersecting Pacific Ocean biomes detected by protein biomarkers. *Science* **345**, 1173–1177 (2014).

31. U. Guyet, N. A. Nguyen, H. Doré, J. Haguait, J. Pittera, M. Conan, M. Ratin, E. Corre, G. le Corguillé, L. Brillet-Guéguen, M. Hoebeke, C. Six, C. Steglich, A. Siegel, D. Eveillard, F. Partensky, L. Garczarek, Synergic effects of temperature and irradiance on the physiology of the marine *Synechococcus* strain WH7803. *Front. Microbiol.* **11**, 1707 (2020).

32. J. R. Casey, A. Mardinoglu, J. Nielsen, D. M. Karl, Adaptive evolution of phosphorus metabolism in *Prochlorococcus*. *mSystems* **1**, e00065-16 (2016).

33. S. Jagadevan, A. Banerjee, C. Banerjee, C. Guria, R. Tiwari, M. Baweja, P. Shukla, Recent developments in synthetic biology and metabolic engineering in microalgae towards biofuel production. *Biotechnol. Biofuels* **11**, 185 (2018).

34. E. Forchielli, D. Sher, D. Segrè, Metabolic phenotyping of marine heterotrophs on refactored media reveals diverse metabolic adaptations and lifestyle strategies. *mSystems* **7**, e00070–22 (2022).

35. R. T. Letscher, J. K. Moore, Preferential remineralization of dissolved organic phosphorus and non-Redfield DOM dynamics in the global ocean: Impacts on marine productivity, nitrogen fixation, and carbon export. *Global Biogeochem. Cycles* **29**, 325–340 (2015).

36. Y. Liu, P. Debeljak, M. Rembauville, S. Blain, I. Obernosterer, Diatoms shape the biogeography of heterotrophic prokaryotes in early spring in the Southern Ocean. *Environ. Microbiol.* **21**, 1452–1465 (2019).

37. S. Dutkiewicz, P. Cermeno, O. Jahn, M. J. Follows, A. E. Hickman, D. A. A. Taniguchi, B. A. Ward, Dimensions of marine phytoplankton diversity. *Biogeosciences* **17**, 609–634 (2020).

38. F. Vincent, C. Bowler, Diatoms are selective segregators in global ocean planktonic communities. *mSystems* **5**, e00444-19 (2020).

39. D. Tilman, J. Knops, D. Wedin, P. Reich, M. Ritchie, E. Siemann, The influence of functional diversity and composition on ecosystem processes. *Science* **277**, 1300–1302 (1997).

40. N. S. Garcia, M. Du, M. Guindani, M. R. Mc Ilvin, D. M. Moran, M. A. Saito, A. C. Martiny, Proteome trait regulation of marine *Synechococcus* elemental stoichiometry under global change. *ISME J.* **18**, wrae046 (2024).

41. M. Royo-Llonch, P. Sánchez, C. Ruiz-González, G. Salazar, C. Pedrós-Alió, M. Sebastián, K. Labadie, L. Paoli, F. M. Ibarbalz, L. Zinger, B. Churcheward, Tara Oceans Coordinators, M. Babin, P. Bork, E. Boss, G. Cochrane, C. de Vargas, G. Gorsky, N. Grimsley, L. Guidi, P. Hingamp, D. Iudicone, O. Jaillon, S. Kandels, F. Not, H. Ogata, S. Pesant, N. Poulton, J. Raes, C. Sardet, S. Speich, L. Setmmann, M. B. Sullivan, S. Chaffron, D. Eveillard, E. Karsenti, S. Sunagawa, P. Wincker, L. Karp-Boss, C. Bowler, S. G.

Acinas, Compendium of 530 metagenome-assembled bacterial and archaeal genomes from the polar Arctic Ocean. *Nat. Microbiol.* **6**, 1561–1574 (2021).

42. N. R. Cohen, M. R. McIlvin, D. M. Moran, N. A. Held, J. K. Saunders, N. J. Hawco, M. Brosnahan, G. R. DiTullio, C. Lamborg, J. P. McCrow, C. L. Dupont, A. E. Allen, M. A. Saito Dinoflagellates alter their carbon and nutrient metabolic strategies across environmental gradients in the central Pacific Ocean. *Nat. Microbiol.* **6**, 173–186 (2021).

43. N. A. Held, J. B. Waterbury, E. A. Webb, R. M. Kellogg, M. R. McIlvin, M. Jakuba, F. W. Valois, D. M. Moran, K. M. Sutherland, M. A. Saito, Dynamic diel proteome and daytime nitrogenase activity supports buoyancy in the cyanobacterium *Trichodesmium*. *Nat. Microbiol.* **7**, 300–311 (2022).

44. C. Pál, B. Papp, Evolution of complex adaptations in molecular systems. *Nat. Ecol. Evol.* **1**, 1084–1092 (2017).

45. B. A. Ward, S. Collins, S. Dutkiewicz, S. Gibbs P. Bown, A. Ridgwell, B. Sauterey, J. D. Wilson, A. Oschlies, Considering the role of adaptive evolution in models of the ocean and climate system. *J. Adv. Model. Earth Syst.* **11**, 3343–3361 (2019).

46. T. J. Browning, E. P. Achterberg, I. Rapp, A. Engel, E. M. Bertrand, A. Tagliabue, C. M. Moore Nutrient co-limitation at the boundary of an oceanic gyre. *Nature* **551**, 242–246 (2017).

47. P. Falkowski, T. Fenchel, E. Delong, The microbial engines that drive Earth’s biogeochemical cycles. *Science* **320**, 1034–1039 (2008).

48. N. D. Price, J. L. Reed, B. O. Palsson, Genome-scale models of microbial cells: Evaluating the consequences of constraints. *Nat. Rev. Microbiol.* **2**, 886–897 (2004).

49. A. Löhne, B. Weißing, Equivalence between polyhedral projection, multiple objective linear programming and vector linear programming. *Math. Methods Oper. Res.* **84**, 411–426 (2016).

50. R. W. Eppley, Temperature and phytoplankton growth in the sea. *Fish. Bull.*. **70**, 1063–1085 (1972).

51. U. B. Singh, A. S. Ahluwalia, Microalgae: A promising tool for carbon sequestration. *Mitig. Adapt. Strateg. Glob. Change* **18**, 73–95 (2013).

52. P. M. Berube, S. J. Biller, A. G. Kent, J. W. Berta-Thompson, S. E. Roggensack, K. H. Roache-Johnson, M. Ackerman, L. R. Moore, J. D. Meisel, D. Sher, L. R. Thompson, L. Campbell, A. C. Martiny, S. W. Chisholm, Physiology and evolution of nitrate acquisition in *Prochlorococcus*. *ISME J.* **9**, 1195–1207 (2015).

53. I. Thiele, B. O. Palsson, A protocol for generating a high-quality genome-scale metabolic reconstruction. *Nat. Protoc.* **5**, 93–121 (2010).

54. A. Varma, B. O. Palsson, Metabolic flux balancing: Basic concepts, scientific and practical use. *Nat. Biotechnol.* **12**, 994–998 (1994).

55. S. Srinivasan, W. R. Cluett, R. Mahadevan, Constructing kinetic models of metabolism at genome-scales: A review. *Biotechnol. J.* **10**, 1345–1359 (2015).

56. H. Doré, U. Guyet, J. Leconte, G. K. Farrant, B. Alric, M. Ratin, M. Ostrowski, M. Ferrieux, L. Brillet-Guéguen, M. Hoebeke, J. Siltanen, G. L. Corguillé, E. Corre, P. Wincker, D. J. Scanlan, D. Eveillard, F. Partensky, L. Garczarek, Differential global distribution of marine picocyanobacteria gene clusters reveals distinct niche-related adaptive strategies. *ISME J.* **17**, 720–732 (2023).

57. A. Larhlimi, S. Blachon, J. Selbig, Z. Nikoloski, Robustness of metabolic networks: A review of existing definitions. *Biosystems* **106**, 1–8 (2011).

58. H. Kitano, Biological robustness. *Nat. Rev. Genet.* **5**, 826–837 (2004).

59. M. P. Gerstl, S. Klamt, C. Jungreuthmayer, J. Zanghellini, Exact quantification of cellular robustness in genome-scale metabolic networks. *Bioinformatics* **32**, 730–737 (2016).

60. C. Kuhlisch, A. Shemi, N. Barak-Gavish, D. Schatz, A. Vardi, Algal blooms in the ocean: Hot spots for chemically mediated microbial interactions. *Nat. Rev. Microbiol.* **22**, 138–154 (2024).

61. R. J. Geider, H. L. MacIntyre, T. M. Kana, Dynamic model of phytoplankton growth and acclimation: Responses of the balanced growth rate and the chlorophyll a:carbon ratio to light, nutrient-limitation and temperature. *Oceanogr. Lit. Rev.* **9**, 974 (1997).

62. M. Cornec H. Claustre, A. Mignot, L. Guidi, L. Lacour, A. Poteau, F. D'Ortenzio, B. Gentili, C. Schmechtig. Deep chlorophyll maxima in the global ocean: Occurrences, drivers and characteristics. *Glob. Biogeochem. Cycles* **35**, e2020GB006759 (2021).

63. S. J. Biller, P. M. Berube, D. Lindell, S. W. Chisholm, *Prochlorococcus*: The structure and function of collective diversity. *Nat. Rev. Microbiol.* **13**, 13–27 (2015).

64. C. Vernette, N. Henry, J. Lecubin, C. de Vargas, P. Hingamp, M. Lescot, The Ocean Barcode Atlas: A web service to explore the biodiversity and biogeography of marine organisms. *Mol. Ecol. Resour.* **21**, 1347–1358 (2021).

65. N. L. Kettles, S. Kopriva, G. Malin, Insights into the regulation of dmsp synthesis in the diatom thalassiosira pseudonana through apr activity, proteomics and gene expression analyses on cells acclimating to changes in salinity, light and nitrogen. *PLOS ONE* **9** e94795 (2014).

66. H. A. Bullock, H. Luo, W. B. Whitman, Evolution of dimethylsulfoniopropionate metabolism in marine phytoplankton and bacteria. *Front. Microbiol.* **8**, 637 (2017).

67. S. Wang, M. Maltrud, S. Elliott, P. Cameron-Smith, A. Jonko, Influence of dimethyl sulfide on the carbon cycle and biological production. *Biogeochemistry* **138**, 49–68 (2018).
